# Self-assembled colloidal gold superparticles to enhance the sensitivity of lateral flow immunoassays with sandwich format

**DOI:** 10.7150/thno.42364

**Published:** 2020-02-19

**Authors:** Xirui Chen, Yuankui Leng, Liangwen Hao, Hong Duan, Jing Yuan, Wenjing Zhang, Xiaolin Huang, Yonghua Xiong

**Affiliations:** 1 State Key Laboratory of Food Science and Technology, Nanchang University, Nanchang 330047, P. R. China;; 2 School of Food Science and Technology, Nanchang University, Nanchang 330047, P. R. China;; 3 Jiangxi-OAI Joint Research Institute, Nanchang University, Nanchang 330047, P. R. China.

**Keywords:** gold nanoparticle, lateral flow immunoassay, self-assembly, colloidal gold superparticle, sensitivity

## Abstract

**Background:** Traditional lateral flow immunoassay (LFIA) based on 20-40 nm gold nanoparticles (AuNPs) as signal reporter always suffers from relatively low detection sensitivity due to its insufficient brightness, severely restricting its wide-ranging application in the detection of target analytes with trace concentration.

**Methods:** To address this problem, the self-assembled colloidal gold superparticles (GSPs) were synthesized as an improved absorption-dominated labeling probe for improving the sensitivity of sandwich LFIA. Five kinds of GSPs with the size ranging from 100 nm to 400 nm were synthesized by embedding hydrophobic AuNPs of size 12 nm as building blocks into the polymer nanobeads. The as-prepared GSPs were suggested as novel labeling probes of LFIA. The effects of the size of assembled GSPs on the sensitivity of sandwich LFIA was assessed, and the detection performance of GSPs-LFIA was further compared with traditional AuNPs-LFIA.

**Results:** The resultant GSPs showed extremely high light absorption but very low light scattering, which favor the absorption-dominated signal output in LFIA. Among them, the GSP_270_-LFIA (size 270 nm) exhibits the highest sensitivity for human chorionic gonadotropin and hepatitis B surface antigen detection in real serum sample, which are approximate 39.79- and 13.8-fold higher than that of traditional AuNP_40_-LFIA.

**Conclusions:** The proposed research demonstrated that the current GSPs can provide an ultrasensitive and quantitative detection for disease biomarkers in real serum samples as promising reporters of sandwich LFIA platform.

## Introduction

Point-of-care (POC) diagnostic devices are integral in rapid diagnostic systems to accelerate prompt on-site diagnosis and treatment decisions and improve the clinical outcomes of patients 1-2. Among the currently available POC diagnostic systems, lateral flow immunoassay (LFIA) is the most promising and attractive POC platform and widely applied in clinical diagnosis, food safety, animal health, and environment monitoring 3, because it provides numerous merits, such as simplicity, rapidity, low cost, visualization, and user-friendliness 4-7. In the past several decades, gold nanoparticles (AuNPs) with sizes of 20-40 nm have dominated the commercialized colorimetric signal probes in LFIA owing to their excellent colloidal stability and characteristic reddish color 8-9. However, conventional AuNP-based LFIA (AuNP-LFIA) often suffers relatively low sensitivity due to its insufficient brightness of 20-40 nm AuNPs, severely restricting its wide-ranging application in the detection of target analytes with trace concentration 10-13. In recent years, various amplification strategies, including noble metal growth 14-17, enzymatic deposition 18, and nanoparticle accumulation 19-20, have been presented to improve the sensitivity of AuNP-LFIA 21. Nevertheless, these methods require complicated chemical synthesis, surface functionalization, and elaborate molecular design, thus compromising the LFIA simplicity, decreasing the reproducibility, and limiting their commercialization. Thus, substantially improving the sensitivity of AuNP-LFIA without increasing complexity still remains to be a huge challenge.

For a classical sandwich LFIA test, enhancing the signal intensity of colorimetric probe and increasing the binding affinity of the complex to captured antibodies on the T line are considered as two main strategies to improve LFIA sensitivity 22-24. Compared with small-sized AuNPs, large-sized AuNPs have stronger optical intensity, which is conducive to increasing LFIA sensitivity. Bischof et al. demonstrated that large-sized AuNPs (100 nm) can allow moderate improvement in the sensitivity compared with 30 nm AuNPs 25. Our previous study also verified that 100 nm AuNPs used as signal reporter can increase the sensitivity of competitive LFIA 26. However, the use of oversized AuNPs (180 nm) as probes in turn decreases LFIA sensitivity despite their higher molar extinction coefficient (ε) than 100 nm. Several possible reasons are responsible for reduced sensitivity. On the one hand, when the target concentration approaches the limit of detection (LOD), each AuNP probe usually combines one or several analytes because the AuNP probe content is far higher than that of the analyte. Therefore, the complex of large-sized AuNPs and analyte should embody a weak binding affinity to captured antibodies at the T line because of the low diffusivity of large-sized AuNPs on the nitrocellulose (NC) membrane, thereby causing poor LFIA sensitivity 27. On the other hand, the extinction efficiencies (*Q*_ext_) of AuNPs consists of the adsorption efficiencies (*Q*_abs_) and the scattering efficiencies (*Q*_sca_) 28. However, when the size of AuNPs exceeds 80 nm, the *Q*_ext_ of AuNPs mainly contributes to the increase of *Q*_sca_, whereas *Q*_abs_ changes slightly 29. Previous work implied that the light absorption rather than scattering of AuNPs dominated the signal readout on the NC membrane 30. Thus, a rapidly increasing localized surface plasmon resonance (LSPR) signal of large AuNPs (100 nm) ensures enhanced sensitivity, whereas a further increase in AuNP size (180 nm) decreases AuNP-LFIA sensitivity despite their exceptional *Q*_ext_. In brief, large AuNPs can moderately enhance the sensitivity, whereas overlarge AuNPs reduce the sensitivity due to their stronger light scattering and lower diffusivity on the NC membrane.

Recent theoretical modeling analysis showcases that when numerous isolated AuNPs are assembled together, the total light absorption will be greatly increased for improved LSPR transduction because of the collective molar extinctions of AuNPs 31. The emulsion-based self-assembly strategy represents the most promising route for constructing AuNP superstructures. Various AuNP assemblies, such as nanoaggregates 32-35, nanoclusters 36-37, and nanovesicles 31, have been fabricated through the hydrophobic interaction between stabilizers and surfactants in the oil-in-water emulsion system 38-39. The obtained Au superstructures show closely packed nanocrystal configurations and unexpected physicochemical and optical properties different from individual AuNPs, facilitating their wide applications in biosensing, bioimaging, drug delivery, and theranostics 40. However, most reported AuNP assemblies exhibit strong plasmonic coupling between two or more AuNPs, causing evident red shifts in LSPR absorption with the color changing from wine red to bluish violet. As a result, such AuNP tonality is not conducive to confident naked-eye detection. Plasmonic coupling is associated with interparticle gaps between AuNPs within the assemblies, and with increasing the interparticle distance, the plasmonic coupling weakens or disappears. Consequently, AuNP assemblies display similar LSPR absorption and color but stronger absorbance relative to the isolated AuNPs, thereby enabling increased sensitivity.

**Figure 1:**
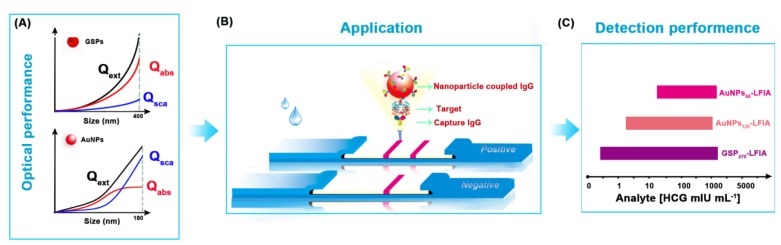
Schematic illustration of GSP-LFIA and AuNP-LFIA. (A) The theoretical Optical performance of GSPs and AuNPs; (B)The illustration of sandwich LFIA platform; (C) The detection sensitivity of GSP-LFIA and AuNP-LFIA.

Herein, we report the successful synthesis of assembled colloidal gold superparticles (GSPs) and demonstrate their potential as an improved absorption-dominated labeling probe for the sensitive and quantitative detection of disease biomarkers on the sandwich LFIA platform (**Figure 1**). The synthetic strategy involved the self-assembly of small-sized hydrophobic AuNPs into large GSPs. We specifically synthesized oleylamine-capped hydrophobic AuNPs with size ~12 nm as building blocks for three reasons: i) such sized AuNPs show relatively strong light absorption with extremely weak light scattering; ii) hydrophobic oleylamine can drive AuNP assembly through hydrophobic interaction in a microemulsion system; iii) the oleylamine surface of AuNPs can prevent them from direct contact during the self-assembly to avoid undesired plasmonic coupling. To further weaken the plasmonic coupling, we introduced poly(maleicanhydride-alt-1-octadecene) (PMAO) as a polymer layer to increase interparticle gap and provide carboxyl groups for antibody functionalization. Subsequently, the assembled GSPs were used as the signal reporter of LFIA for human chorionic gonadotropin (HCG) and hepatitis B surface antigen (HBsAg) detection. The impact of the size and optical properties of assembled GSPs on the sensitivity of sandwich LFIA was evaluated, and the detection performance of GSPs-LFIA was further compared with traditional AuNPs-LFIA.

## Results and Discussion

### Synthesis and Structural Characterization of GSPs

**Figure 2:**
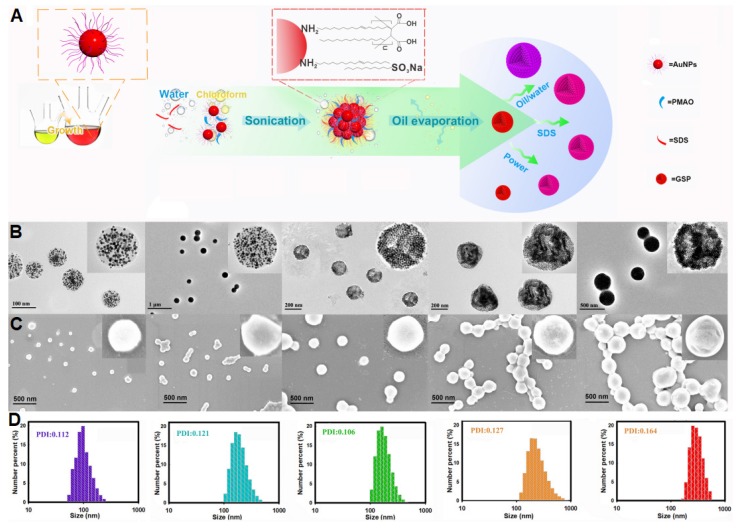
(A) Illustration of the synthetic strategy for GSPs by the microemulsion-based self-assembly process. Characterization of five different-sized GSPs. (B) TEM images. (C) SEM images. (D) DLS analysis. For Figure 2B-D, from left to right: GSP_100_, GSP_160_, GSP_200_, GSP_270_, and GSP_400,_ respectively.

**Figure 2A** illustrates the synthetic strategy for GSPs by the microemulsion-based self-assembly process. Oleylamine-capped hydrophobic AuNPs with size of 12 nm were used to demonstrate the successful formation of the assembled GSPs (**Figure S1**). In a typical procedure, a solution of hydrophobic AuNPs in toluene with desired amounts of PMAO was added into the SDS water solution, followed by ultrasonic emulsification. After the evaporation of toluene, the self-assembled GSPs were obtained. The precisely controlled GSP size was easily achieved by changing the SDS amount, volume ratio of oil/water, and ultrasonic power (**Table S1**). These synthesized GSPs were then characterized by TEM, SEM, and DLS. The TEM images in **Figure 2B** reveal that the hydrophobic AuNPs were successfully assembled into spherical ensembles of closely attached nanoparticles with precise control over GSP size. The magnified TEM image of a single GSP is presented in the inset of **Figure 2B**, where numerous, individual AuNPs with size of 12 nm were tightly dispersed in a spherical polymer matrix. When GSP size was increased from 100 nm to 400 nm, the encapsulation numbers of hydrophobic AuNPs remarkably increased from 639 to 30,908 (**Figure 2B**, the detailed calculation was described in Supplementary Information). SEM observation (**Figure 2C**) showed regular spherical structures of GSPs with densely packed AuNPs visible at high magnification, indicating high loading capacity and the homogeneous distribution of AuNPs (brighter spots relative to the surrounding polymer matrix) throughout a single GSP nanosphere. The size distribution and colloidal dispersion of the synthetic GSPs were further confirmed by DLS (**Figure 2D**). The results indicated that the hydrodynamic diameters of the GSPs ranged from 100 nm to 400 nm with a relatively narrow size distribution, which was consistent with the results obtained from TEM and SEM. Furthermore, all polydispersity indices of the assembled GSPs as measured by DLS were less than 0.2, ensuring their synthesis repeatability. These findings demonstrated that the as-prepared GSPs possessed uniform morphological structures and excellent monodispersity, making them well-suited labeling probes for LFIA development. The precise control of GSP size using this current self-assembly synthesis strategy provides an ideal platform for evaluating the effect of particle size on the sensitivity in LFIA. Four different sized citrate modified-AuNPs (40, 80, 120, and 180 nm) were prepared by using the seed growth method (**Figure S2**) and then applied as labeled probes to enable direct comparison with GSPs in the optical properties and detection performance.

### Optical Properties of GSPs

**Figure 3:**
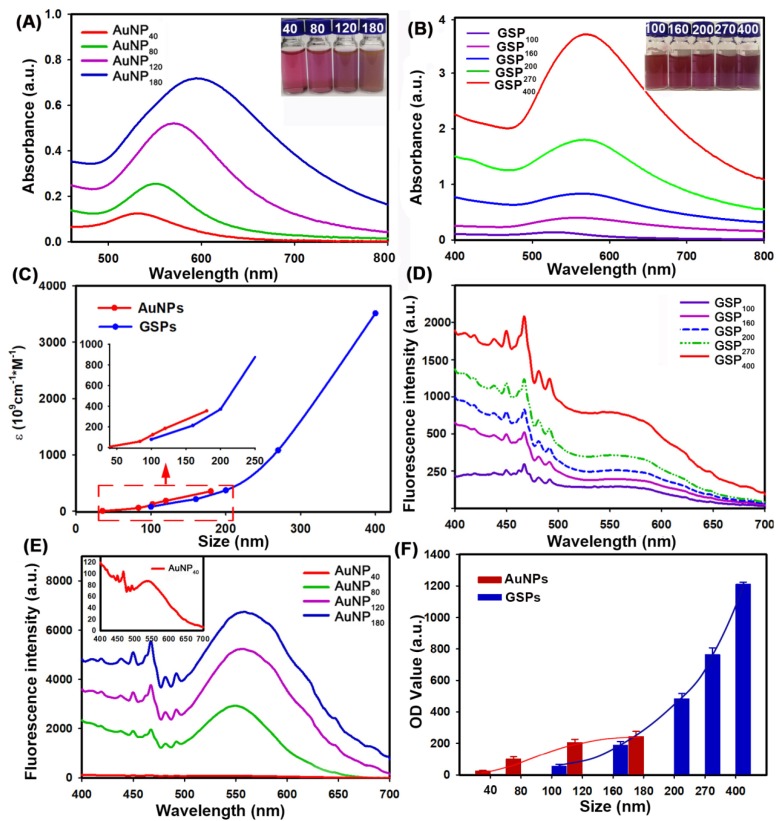
Characterization of the optical performance of citrate modified-AuNPs and the resultant GSPs. (A) and (B) UV-Vis spectrum analysis of citrate modified-AuNP and GSP samples at the same particle concentration of 1.17 pM. The maximum absorption peaks for AuNP_40_, AuNP_80_, AuNP_120_, and AuNP_180_ centered at 527, 556, 572, and 598 nm, and the maximum absorption peaks for GSP_100_, GSP_160_, GSP_200_, GSP_270_, and GSP_400_ were at 532, 538, 543, 551, and 556 nm. (C) Relationship between the ε values and the size of AuNP or GSP. (D) and (E) Light scattering intensity (OD_sca_) analysis of citrate modified-AuNPs and GSPs. (F) Comparison of the OD values by spraying the AuNPs or GSPs as the T lines at the same molar concentrations.

The colorimetric signal intensity of the labeling probe is one of the most crucial elements in LFIA because it determines signal intelligibility and sensitivity 41. Thus, prior to employing them to LFIA, we first estimated the optical properties of the designed GSPs. For comparison, four citrate modified-AuNPs were also investigated. The corresponding UV-Vis absorption spectra obtained from citrate modified-AuNPs and GSP samples at the same particle concentration are displayed in **Figure 3A**-**B**, respectively. As shown in **Figure 3A**, the optical absorbance showed evident enhancement as the size of citrate modified-AuNPs increased from 40 nm to 180 nm. Meanwhile, the maximum absorption peak exhibited a significant red shift from 527 nm to 598 nm with the color of AuNP solution changing from wine red to brick red with increasing AuNP size (inset of **Figure 3A**). By contrast, the resultant GSPs showed similar increased optical absorbance over particle size (**Figure 3B**). However, only a slight red shift from 532 nm to 556 nm was observed with the increase in GSP size from 100 nm to 400 nm. Thus, these synthetic GSPs appeared as obvious red or amaranth color, providing distinct visualization readout (inset of **Figure 3B**), which is beneficial for LFIA. To compare the optical properties, the ε value of the prepared AuNPs and GSPs were estimated and presented in **Figure 3C** (The detailed calculation was described in Supplementary Information). The red and blue lines indicate that the ε values of AuNP and GSP significantly increase with the size of AuNP and GSP increasing. These results suggested that increasing the AuNP or GSP size can improve optical intensity. Notably, the inset in **Figure 3C** indicated that the ε values of GSPs are greater than that of 180 nm AuNPs when the size of GSPs is larger than 200 nm. The significantly enhanced optical signal intensity of the designed GSP nanosphere is the basis for the exceptional sensitivity in LFIA.

As described earlier, the ensemble LSPR absorption spectra were generally evaluated by determining the corresponding *Q*_ext_, including *Q*_abs_ and *Q*_sca_ 42. These values are described in the following equation: *Q*_ext_ = *Q*_abs +_* Q*_sca_. To further characterize the relative contribution of *Q_abs_* and *Q_sca_* within the ensemble *Q*_ext_, we selected four citrate modified-AuNP samples (40-180 nm) and five GSP samples (100-400 nm) to determine the changes in light scattering intensity over particle size under the same concentration. The light scattering spectra of AuNPs and GSPs were performed by synchronous scanning of the excitation and emission monochromators from 300.0 ~ 700.0 nm by using a fluorescence spectrophotometer (Hitachi F-4500, Tokyo, Japan) 43. The results in **Figure 3D**-**E** showed that light scattering intensity (OD_sca_) of AuNPs or GSPs increased with the increase of AuNP or GSP size, indicating an increased contribution from *Q*_sca_ for the ensemble *Q*_ext_ with particle size. Nevertheless, the OD_sca_ value of AuNPs increased steeply as the AuNP size was increased, resulting in an evident change from absorption-dominated for small-sized AuNPs to scattering-dominated for large-sized AuNPs, which was accordance with a previous report 42. By contrast, the OD_sca_ of GSPs increased slightly with increasing GSP size, suggesting that absorption dominated the overall extinction spectra for GSPs. We also found that the OD_sca_ value of the customized GSPs was lower than that of AuNPs at similar particle size. For example, GSP_200_ had a similar size with AuNP_180_), whereas the OD_sca_ value of GSP_200_ at 550 nm was only one-twentieth (312/6752) of that of AuNP_180_. Even though the GSP size increased to 270 nm, the OD_sca_ was one-fifteenth (464/6752) of that of AuNP_180_. From the above discussion, the designed GSPs exhibited significantly increased absorption with slightly elevated scattering. To further explain the contribution of light absorption (OD_abs_) and OD_sca_ to the OD value on the T line, we sprayed all resultant AuNPs and GSPs at the same molar concentrations onto the NC membrane as T line, and the corresponding OD values were then recorded with a strip reader. To suppress the coffee ring effect during spraying, the AuNPs and GSPs were mixed with 1% BSA and 10% glycerine. The results in **Figure 3F** indicated that the OD values of the T line gradually increased with increasing AuNP size from 40 nm to 120 nm and then reached a balance with continuous increase to 180 nm. This result indicated that although AuNP_180_ possessed far higher ε value than AuNP_120_, no significant difference in OD value at the T line was observed between the two AuNPs, further verifying that the scattering-dominated AuNP_180_ could only provide a very small contribution to the absorption-associated OD value in LFIA. Interestingly, we found that as the GSP size increased from 100 nm to 400 nm, the OD values at the T line presented an exponential increase without balance, which was accordance with the size-dependent increase in the optical absorbance of GSPs. This finding illustrated that the assembled GSPs provided remarkably enhanced optical absorption and could serve as amplified colorimetric labels for improving LFIA sensitivity. Further characterizations of the GSP_270_ against pH value, dispersion in solution and long-term stability were conducted and the results in **Figure S3** revealed excellent optical stability, high colloidal and long-term stability of our GSP_270_ nanosphere, which favors potential biological applications. In summary, these results verified that the self-assembly strategy for individual small-sized AuNPs to assemble into large-sized AuNP superstructures is promising for designing absorption-dominated nanoparticle labels with high colloidal and optical stability. This significantly enhanced optical absorbance of the current GSPs is much desired for highly sensitive absorption-based detection methods.

### Highly Sensitive and Quantitative HCG Detection in Serum via GSP-LFIA Strip

Encouraged by the improved optical property, we investigated the feasibility of the customized GSPs in highly sensitive colorimetric detection. In this case, the GSPs were utilized as visual contrast labels in LFIA strip. HCG, a key diagnostic marker of pregnancy and an important risk biomarker of certain diseases, was selected as a model analyte 44. For direct comparison, we benchmarked the performance of GSPs in LFIA against AuNPs with the same set of antibodies and materials. The GSP-LFIA or AuNP-LFIA strip design shares the classical sandwich LFIA construction. A series of important factors that affect the analytical sensitivity were optimized prior to the GSP-LFIA and AuNP-LFIA strip development (**Figures S4** to** S15**), and the details are summarized in **Table S2**. Under the optimal conditions, we systematically compared the detection performance of GSP-LFIA and AuNP-LFIA strips, including a qualitative assay using the naked eyes and a quantitative assay with a commercial strip reader. For HCG qualitative assay, the visual LOD (vLOD), defined as the lowest HCG concentrations for generating a visible red band at the T line, was evaluated 45. **Figure S16A** illustrates that when the AuNP size was increased from 40 nm to 120 nm, the vLOD value decreased from 39 mIU/mL for AuNP_40_ to 7.8 mIU/mL for AuNP_80_ and further to 1.95 mIU/mL for AuNP_120_, a 5-fold and 20-fold improvement for AuNP_80_ and AuNP_120_ in the detection sensitivity compared with that of AuNP_40_, respectively. This result suggested that large-sized AuNPs could indeed increase LFIA sensitivity. One possible reason is that large-sized AuNPs possess stronger optical signal intensity than small-sized AuNPs. This finding agreed well with the results obtained by Bischof et al 25. However, we found that when AuNP size was further increased to 180 nm, the vLOD instead increased to 3.9 mIU/mL, showing a 2-fold reduction in the LFIA sensitivity compared with that of AuNP_120_. For HCG quantitation, the concentration-dependent change in the OD value at the T line was recorded by a strip reader under different HCG concentrations. The OD values at the C lines were also collected as a reference to allow a more reliable detection via the OD_T_/OD_C_ values. The calibration curves were obtained by plotting the OD_T_/OD_C_ values of the HCG standard solutions in artificial serum against HCG concentrations. The results demonstrated an excellent power correlation between the OD_T_/OD_C_ values and target concentrations (**Figure S16B**-**E**). Accordingly, the corresponding LOD values (defined as the lowest HCG concentration for a detectable OD value on the T line) 46, dynamic detection range, and Hook effect point are summarized in **Table 1**. The results indicated that the AuNP_120_-LFIA strip exhibited the lowest LOD value of 0.97 mIU/mL, which was *ca.* 20.1-, 4.02-, and 2.01-fold lower than those of AuNP_40_ (19.5 mIU/mL), AuNP_80_ (3.9 mIU/mL), and AuNP_180_ (1.95 mIU/mL), respectively. The linear detection of AuNP_120_-LFIA strip ranged from 1.9 mIU/mL to 1000 mIU/mL. Thus, we conclude that the LFIA sensitivity increased when AuNP size was increased from 40 nm to 120 nm. However, when AuNP size was further increased to 180 nm, the sensitivity decreased despite the increased optical signal. We speculate that this result may be due to the significantly enhanced *Q*_sca_ rather than* Q*_abs_ for increased *Q*_ext_ of AuNP_180_, conflicting with absorption-dominated signal output of AuNP-LFIA. In addition, the oversized AuNPs (e.g., 180 nm) may cause low AuNP capture at the T line due to the decreased diffusion of the formed complex between AuNP probe and target.

**Figure 4:**
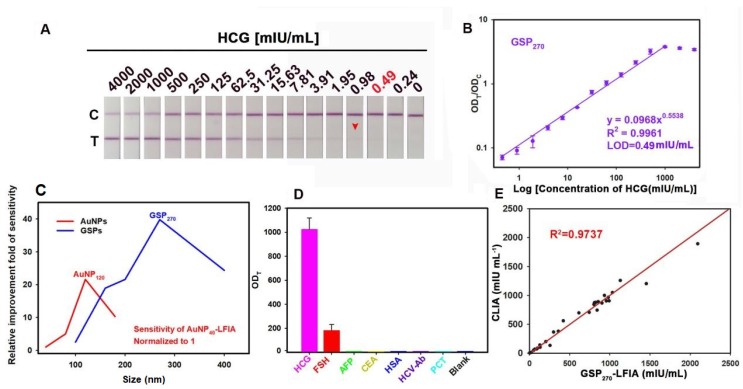
GSP_270_-LFIA test strips for qualitative and quantitative analysis of HCG in serum. (A) Qualitative analysis of HCG by recording the strip prototypes after testing a series of HCG samples with the concentration ranging from 0 mIU/mL to 4000 mIU/mL. (B) Relationship between the OD_T_/OD_C_ value and HCG concentration from 0 mIU/mL to 4000 mIU/mL, in which an excellent correlation for HCG determination was observed with a target concentration of 0.49 mIU/mL to 2000 mIU/mL. (C) Comparison of the detection sensitivities of AuNP- and GSP-LFIA under different particle sizes. The sensitivity of AuNP_40_-LFIA (19.5 mIU/mL) is normalized to 1, and other LFIA strips are normalized to the improvement folds relative to AuNP_40_-LFIA. (D) Evaluation of the specificity by measuring other common serum protein biomarkers with our proposed GSP_270_-LFIA. (E) Correlation analysis of the detection results between the GSP_270_-LFIA and CLIA methods in 30 human serum samples with HCG concentrations from 0.67 mIU/mL to 2000 mIU/mL.

As previously described, when small-sized AuNPs were assembled into large GSPs, the ensemble optical intensity showed a significant increase, especially *Q_abs_*, which contributes to highly sensitive LFIA with GSPs as visual labels. **Figure 4A** and **Figure S17A** show the strip prototype responding to varying target concentrations using five sized GSPs as reporter. As the GSP size increased from 100 nm to 270 nm, the vLOD decreased from 7.8 mIU/mL to 0.98 mIU/mL. With further increase of GSP size to 400 nm, the vLOD increased to 1.9 mIU/mL. Given these results, we speculate that the excessively low diffusivity of over-sized GNPs could settle within the pores of the NC membrane and then result in a remarkably nonspecific binding of the GSP probe on the T line. Consequently, the lowest vLOD of 0.98 mIU/mL was achieved using GSP_270_ as visual labels. Further analysis of the OD_T_/OD_C_ value indicated that five GSPs could provide linear detection against HCG concentration, and the results are shown in **Figure 4B** and **Figure S17B**-**E**. Following the corresponding standard curves, the LOD values, the dynamic detection range, and the Hook effect point are summarized in **Table 1**. The results showed that the lowest LOD value of 0.49 mIU/mL was obtained using GSP_270_ as LFIA label, which was *ca.* 39.79-fold lower than that of conventional AuNP_40_-LFIA strip (LOD: 19.5 mIU/mL) and 1.98-fold lower than that of AuNP_120_-LFIA strip (LOD: 0.97 mIU/mL). Notably, although the GSP_400_ exhibited higher *Q*_abs_ than GSP_270_, the LOD of 1.8 mIU/mL using GSP_400_ as a label was *ca.* 3.67-fold higher than that of GSP_270_-LFIA strips. The above results demonstrated that when the GSP size ranged from 100 nm to 400 nm, *Q*_ext_ was largely magnified, especially *Q*_abs_, which is beneficial for improving the LFIA sensitivity using large-sized GSPs as probes. However, only the GSPs with the appropriate size (e.g., 270 nm) could produce the highest sensitivity in the sandwich LFIA. The over-sized GSPs (e.g., 400 nm) settled in the membrane pores and generated a high background residue at the T zone due to their slow diffusion rates, thereby deteriorating LFIA sensitivity. As shown in **Figure S18**, evident background bands at the T zones were observed when we used the GSP_400_-LFIA strip to detect 10 blank samples. However, no background signal was seen with the other four GSP-LFIA strips, further confirming that 400 nm partly settled in the test area of NC membrane to form background value even in the absence of targets. As displayed in **Figure 4C**, large AuNPs (e.g., AuNP_120_) and GSPs (e.g., GSP_270_) were capable of ensuring 20.1- and 39.79-fold improvements in LFIA sensitivity compared with AuNP_40_. However, overlarge particle size resulted in reduced LFIA sensitivity. In addition, the colloidal chemical stability of AuNP_120_ should be considered before large-scale promotion. By contrast, given their high colloidal stability, GSP_270_ is well-suited as an improved LFIA label to improving the sensitivity of traditional AuNP_40_-LFIA. Thus, GSP_270_ was selected as the LFIA signal reporter for the succeeding evaluation. The selectivity of the developed GSP_270_-LFIA strip was determined using several common serum proteins, including follicle-stimulating hormone (FSH), alpha fetoprotein (AFP), carcinoembryonic antigen (CEA), human serum albumin (HSA), hepatitis C virus antibody (HCV-Ab), and procalcitonin (PCT). **Figure 4D** reveals that these non-target proteins could not induce evident increase in the OD_T_/OD_C_ value, whereas the distinct elevation of OD_T_/OD_C_ was achieved in the presence of HCG compared with the blank control, suggesting the excellent discrimination capability of our GSP_270_-LFIA for HCG against other interfering components. Notably, FSH exhibited a small increase in the OD_T_/OD_C_ value, indicating a weak cross-reaction with FSH for HCG detection because of their analogous structures. Given their outstanding advantages of simplicity, convenience, rapidity, sensitivity, and specificity, our GSP_270_-LFIA strip was further extended to clinical diagnostics of HCG in actual serum. Correlation analysis was conducted between the GSP_270_-LFIA strip and the clinically accepted chemiluminescent immunoassay (CLIA) in 30 serum samples. **Figure 4E** presents that when the HCG concentration was increased from 0.67 mIU/mL to 2000 mIU/mL, a good linear correlation between the two methods was obtained with an R^2^ of 0.9737, indicating the robustness of the GSP_270_-LFIA strip in practical biomarker diagnosis from complex biological matrix.

### Highly Sensitive and Quantitative HBsAg Detection in Serum with GSP-LFIA Strip

**Figure 5:**
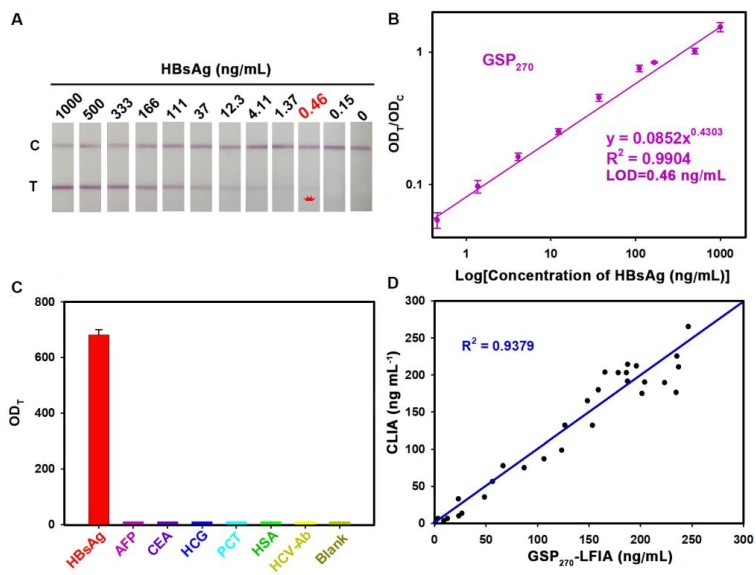
GSP_270_-LFIA test strips for qualitative and quantitative analysis of HBsAg in serum. (A) Qualitative analysis of HBsAg by recording the strip prototypes after testing a series of HBsAg samples with the concentration ranging from 0 ng/mL to 1000 ng/mL. (B) Excellent correlation between the OD_T_/OD_C_ value and HBsAg concentration from 0.46 ng/mL to 1000 ng/mL. (C) Selectivity evaluation of our method by detecting the response against other common serum protein biomarkers with our GSP_270_-LFIA. (D) Correlation analysis of the detection results between the GSP_270_-LFIA strip and the clinically well-accepted CLIA kits in 45 human serum samples with HBsAg concentrations of 0.46 ng/mL to 256 ng/mL.

To demonstrate the versatility of our strategy, we further extended the GSP-LFIA strip for the sensitive detection of HBsAg, an important serological biomarker for hepatitis B virus infection diagnosis 47. In this case, GSP_270_ was selected as visual label for the fabrication of GSP_270_-LFIA strip because GSP_270_ possesses large optical absorbance and good diffusivity on the NC membrane. For direct comparison, the AuNP_40_-LFIA strip was developed at the same time. Several key parameters that influence the sensitivity of AuNP_40_-LFIA and GSP_270_-LFIA strip were systematically investigated and optimized (**Figures S19** and **S20**). Under the optimum conditions, a series of HBsAg standard solutions in artificial serum with target concentration ranging from 0 ng/mL to 1000 ng/mL were tested simultaneously using AuNP_40_-and GSP_270_-LFIA strip. The strip photographs obtained at different HBsAg concentrations are shown in **Figure 5A**. The results indicated that the vLOD of GSP_270_-LFIA strip for HBsAg reached up to 0.46 ng/mL, which was *ca.* 13.8-fold lower than that of AuNP_40_-LFIA (6.2 ng/mL, **Figure S21**). **Figure 5B** presents that the OD_T_/OD_C_ value increased as the HBsAg concentration increased, and an excellent linear relationship between them was observed from 0.46 ng/mL to 1000 ng/mL with an R^2^ of 0.9902. The LOD of current GSP_270_-LFIA strip for HBsAg was 0.46 ng/mL. The specificity analysis in **Figure 5C** suggested the excellent selectivity of this GSP_270_-LFIA strip for HBsAg against other common serum interferences, including AFP, CEA, HCG, PCT, HCV-Ab, and HSA. Precision estimation of our proposed method was performed by calculating the intra- and inter-assay recoveries and coefficients of variation (CV) of five HBsAg-spiked serum samples with HBsAg concentrations of 10, 20, 100, 200, and 500 ng/mL. As displayed in **Table 2**, the average recoveries for intra- and inter-assay changed from 79.53% to 110.58%, with the CV variation from 2.01% to 13.41%, demonstrating an acceptable precision for HBsAg quantification. Considering its excellent sensitivity and specificity, the developed GSP_270_-LFIA was further applied for clinical HBsAg diagnosis in actual serum. Forty-five HBsAg-positive serum samples from clinically diagnosed hepatitis B patients and ten HBsAg-negative serum samples from healthy volunteers were synchronously analyzed by three methods, including GSP_270_-LFIA, AuNP_40_-LFIA, and the clinically well-accepted CLIA kits. Results in **Table S3** reveal that no false positive and false negative results were obtained for GSP_270_-LFIA compared with CLIA, and a high linear dependence with R^2^ of 0.9379 was observed between the two approaches (**Figure 5D**). However, the false negative results appeared thrice in testing HBsAg-positive serum samples using AuNP_40_-LFIA because their concentrations were below the LOD value of AuNP_40_-LFIA (6.2 ng/mL). The above results indicated that the GSP_270_-LFIA achieved comparable performance with the laboratory-based CLIA method in terms of detection sensitivity and accuracy but better than that of traditional AuNP_40_-LFIA.

## Conclusion

We successfully synthesized a novel self-assembled GSP by using hydrophobic AuNPs as building blocks via emulsion-based self-assembly method. The obtained GSPs consisted of numerous, individual small-sized densely packed AuNPs and their size could be easily controlled by simply adjusting the synthesis conditions. The synthesized GSPs were characterized with size-dependent exponential increases in *Q*_ext_, especially the *Q*_abs_, which contributes to enhancing the detection sensitivity of absorption-dominated LFIA method. Notably, the light scattering of current GSPs slightly increased with increased GSP size, in contrast with conventional AuNPs with markedly enhanced light scattering over size. Using GSPs as enhanced optical labels, an ultrasensitive and quantitative determination of two common disease biomarkers, including HCG and HBsAg, was achieved on a sandwich GSP_270_-LFIA platform. The sensitivities for HCG and HBsAg in serum were approximate 39.79- and 13.8-fold higher than that of traditional AuNP_40_-LFIA, respectively. In addition, the sensitivity of our method is comparable to or better than those of other reported gold-based immunoassay methods (**Table S4**). By contrast, overlarge GSPs of size > 400 nm reduced the LFIA sensitivity because of their excessively low diffusivity and rapid sedimentation in the membrane pores. In summary, this work provided a novel strategy to overcome the limitation of inherent weak absorption and size-dependent scattering in AuNPs and break the LOD barrier to extend the application of LFIA in trace detection.

## Experimental Section

### Materials and Reagent

Oleylamine,sodium dodecyl sulfonate (SDS), gold(III) chloride hydrate, trisodium citrate (Na_3_C_6_H_5_O_7_•2H_2_O), bovine serum albumin (BSA), 1-ethyl-3-(3-dimethylaminopropyl) carbodiimide (EDC), PMAO (MW=30000~50000 Da) were purchased from Sigma-Aldrich (St. Louis, MO, USA). The sample pad, the absorbent pad, and the NC membrane were provided by Wuxi Zodolabs Biotech Co., Ltd. (Jiangsu, China). Goat anti-mouse antibody, Human Chorionic Gonadotropin (HCG), anti-HCG-α monoclonal antibody (anti-HCG-α mAb), and anti-HCG-β monoclonal antibody (anti-HCG-β mAb) were obtained from Chongqing Xinyuanjiahe Biotechnology Inc. (Chongqing, China). Carcinoembryonic antigen (CEA), human alpha fetoprotein (AFP), and procalcitonin (PCT), hepatitis C virus (HCV), bovine serum albumin (BSA), Ovalbumin (OVA), and HCG standard substances were purchased from Wanhua Biochem Products Co., Ltd. (Nanchang, China). Deactivated Hepatitis B Surface Antigen (HBsAg, 2.5 mg/mL), mouse anti-HBsAg monoclonal antibody (anti-HBsAg mAb, 7.2 mg/mL) and goat anti-HBsAg polyclonal antibody (anti-HBsAg pAb, 6.8 mg/mL) were supplied by Jinyuan Jiahe Biotechnology Co., Ltd. (Beijing, China). Other chemicals were of analytical grade and purchased from Sinopharm Chemical Corp. (Shanghai, China). All reagents were used without further purification.

### Characterization

The morphology and structure of the prepared GSPs were investigated using a JEOL JEM 2100 transmission electron microscope (TEM, Tokyo, Japan) and a Hitachi S-4800 scanning electron microscope (SEM, Tokyo, Japan). Dynamic light scattering (DLS) analysis was performed using a Zetasizer Nano-ZEN3700 instrument (Malvern, UK) to determine the size distribution of various GSPs. Ultraviolet-visible (UV-Vis) absorption spectra were obtained using an Amersham Pharmacia Ultrospec 4300 pro UV/visible spectrophotometer (England, UK). Fluorescence spectra were assessed with a Hitachi F-4500 fluorescence spectrophotometer (Tokyo, Japan). A commercial HG-8 strip reader was purchased from Shanghai Huguo Science Instrument Co., Ltd. (Shanghai, China).

### Synthesis of Hydrophobic AuNPs

12 nm hydrophobic AuNPs were prepared via the previously reported method 48. In brief, a mixed solution was prepared by mixing gold(III) chloride hydrate (0.3 mmol) with oleylamine (7.4 mmol) in toluene (1.0 mL). The mixture was then added quickly into a boiling solution of oleylamine (35.3 mmol) dissolved in toluene (49 mL) under magnetic stirring. During the reaction, the solution color immediately changed to bright yellow and then gradually turned into deep red after 10 min. After reaction for 3 h, the hydrophobic AuNPs were precipitated by adding 50 mL of ethyl alcohol and then collected by centrifugation. Finally, the hydrophobic AuNPs were vacuum-dried for 2 h at 37 °C and stored for further use.

### Synthesis of Carboxylated GSPs

270 nm GSPs (GSP_270_) were prepared as described previously with slight modification 49. A toluene (20 μL) solution containing hydrophobic AuNPs (10 mg) and PMAO (0.5 mg) was added into the SDS aqueous solution (5 mg, 250 μL). The formed mixed solution was emulsified by ultrasonication for 2 min under 154 W ultrasonic power. After toluene was evaporated at 60 °C for 2 h, the synthesized GSP_270_ were collected by centrifugation and then re-suspended in a phosphate buffer (PB) solution (0.01 M, pH 10) for 24 h to hydrolyze the anhydride group of PMAO into the carboxyl group. Afterward, the resulting carboxylated GSP_270_ were collected and washed thrice with water via centrifugation. Other sized GSPs were synthesized only by altering the SDS amount, volume ratio of oil/water, and ultrasonic power, as shown in **Table S1**.

### Synthesis of GSP_270_ Probes

GSP_270_ probes were synthesized by the production of amido bond between the carboxyl group of GSP_270_ and the amino group of antibodies (anti-HCG-β-mAb or anti-HBsAg-mAb) in the presence of EDC. In brief, 1 μL of anti-HCG-β-mAb (5.6 mg/mL) or anti-HBsAg-mAb (3 mg/mL) was added into 400 μL of 0.01 M pH 7.0 PB solution containing GSP_270_ (6.25 pM) and EDC (1 μg). After reaction for 90 min at room temperature, BSA (10 mg) was added into the above mixture solution and allowed to react for 1 h to block the unreacted carboxyl group. The resulting GSP_270_ probes were then purified via centrifugation; resuspended PB solution (200 μL, 0.01 M, pH 7.4) containing 25% sucrose, 1% BSA, and 0.1% sodium azide; and stored at 4 °C for subsequent use.

### Preparation of GSP-LFIA Strips

GSP-LFIA strips were prepared according to our previously reported procedure with minor modifications 50. A ZX1000 Dispensing Platform and a CM4000 Guillotine Cutter (BioDot Inc.) were used for strip fabrication. The GSP-LFIA strips comprised four components: a sample pad, an NC membrane, an absorbent pad, and a polyvinyl chloride (PVC) backing card. The NC membrane with test (T) and control (C) lines was prepared by separately spotting anti-HCG-α mAb (2.5 mg/mL, for T line) or anti-HbsAg mAb (3 mg/mL, for T line) and goat anti-mouse IgG (2 mg/mL, for C line) at the density of 0.74 mL/cm with a ZX1000 Dispensing platform. The two lines were positioned at a 6.0 mm interval. The NC membrane was then dried overnight at 37 °C. Finally, the sample pad, NC membrane, and absorbent pad were sequentially assembled onto the PVC backing card with an overlap of ~2 mm between them, cut into strips with a width of 3.9 mm, and packaged in a drying cylinder at ambient temperature for further use. The procedure for preparing AuNP-LFIA strips was the same as that for the GSP-LFIA strips.

### Human Serum Testing with GSP-LFIA Strips

The detection procedure of the GSP-LFIA strips was conducted in accordance with our previous report 51. Approximately 2 μL of GSP_270_ probes was incubated with 68 μL of sample solution for 5 min. The sample mixture was then pipetted into the sample well of strip and moved along the NC membrane to the T and C lines. After reaction for 20 min at room temperature, the prototype images of the GSP-LFIA strips were collected. For quantification, the corresponding optical densities on the T line (OD_T_) and C line (OD_C_) were recorded with a commercial HG-8 strip reader. Positive results following the accumulation of target-bound GSP_270_ immunocomplex were demonstrated by the appearance of red bands at the T and C lines. The coloring of the C line only signified a negative test result.

## Supplementary Material

Supplementary figures and tables.

## Figures and Tables

**Table 1:** Summary of the detection performance of AuNP- and GSP-LFIA strip in detecting HCG.

Labels	Linear range (mIU/mL)	R^2^	LOD (mIU/mL)	Hook-effect (mIU/mL)	Regression equation
AuNP_40_	19.5-1250	0.9978	19.5	>5000	y = 0.0142x^0.6674^
AuNPs_80_	7.8-1000	0.9921	3.90	2000	y = 0.0129x^0.85^
AuNPs_120_	1.9-1000	0.9955	0.97	2000	y = 0.0082x^0.8577^
AuNPs_180_	3.9-1000	0.9912	1.95	2000	y = 0.036x^0.8912^
GSP_100_	15.6-2000	0.9914	7.80	4000	y = 0.0223x^0.6448^
GSP_160_	3.9-2000	0.9949	1.03	4000	y = 0.0772x^0.4695^
GSP_200_	1.9-1000	0.9912	0.90	2000	y = 0.0563x^0.5942^
GSP_270_	0.49-1000	0.9961	0.49	2000	y = 0.0968x^0.5538^
GSP_400_	1.95-2000	0.9989	1.80	8000	y = 0.2156x^0.3802^

**Table 2:** Precision analysis of the developed GSP_270_-LFIA test strip in testing HBsAg-positive serum with concentrations of 10, 50, 100, 200, and 500 ng/mL.

Diluted concentration (ng/mL)	Intra-assay precision^a)^					Inter-assay precision^b)^				
	Recovered concentration		CV (%)	Recovery (%)			Recovered concentration		CV (%)	Recovery (%)
500	491.8±9.89		2.01	98.36			502.89±65.32		12.99	100.58
200	162.83±10.52		6.46	81.42			159.05±16.17		10.16	79.53
100	105.08±8.27		7.87	105.08			110.17±6.98		6.34	110.17
50	43.27±4.72		10.91	86.54			48.89±3.24		6.62	97.78
10	9.02±1.03		11.41	90.20			9.17±1.23		13.41	91.70
										

a) The assay was carried out in triplicates on the same day; b) The assay was performed on three consecutive days.
