# Supplementary material for: Self-assembled colloidal gold superparticles to enhance the sensitivity of lateral flow immunoassays with sandwich format
Source: Theranostics. 2020 Feb 19;10(8):3737–48. doi: 10.7150/thno.42364 (PMC7069069; doi:10.7150/thno.42364)
Supplement: Supplementary file 1 — Supplementary figures and tables. [file thnov10p3737s1.pdf]

## Supporting Information for:

# Self-assembled colloidal gold superparticles to enhance the sensitivity of lateral flow immunoassays with sandwich format

Xirui Chen[a,b‡], Yuankui Leng[a,b‡], Liangwen Hao[a,b], Hong Duan[a,b], Jing Yuan[a,b], Wenjing Zhang[a,b], Xiaolin Huang*[a,b], and Yonghua Xiong*[a,b,c]

[a] State Key Laboratory of Food Science and Technology, Nanchang University, Nanchang 330047, P. R. China;

[b] School of Food Science and Technology, Nanchang University, Nanchang 330047, P. R. China;

[c] Jiangxi-OAI Joint Research Institute, Nanchang University, Nanchang 330047, P. R. China;

[‡]These authors contributed equally to this work.

*Correspondence to:

Dr. Xiaolin Huang and Dr. Yonghua Xiong
State Key Laboratory of Food Science and Technology, and Jiangxi-OAI Joint Research Institute, Nanchang University
Address: 235 Nanjing East Road, Nanchang 330047, P.R. China
Phone: +0086-791-8833-4578. Fax: +0086-791-8833-3708
E-mail: hxl19880503@163.com (X. H.); yhxiongchen@163.com (Y. X.)

## List of content

**Supplementary results** ........ 3
**Figure S1** ........ 4
**Figure S2** ........ 5
**Figure S3** ........ 6
**Figure S4** ........ 7
**Figure S5** ........ 8
**Figure S6** ........ 9
**Figure S7** ........ 10
**Figure S8** ........ 11
**Figure S9** ........ 12
**Figure S10** ........ 13
**Figure S11** ........ 14
**Figure S12** ........ 15
**Figure S13** ........ 16
**Figure S14** ........ 17
**Figure S15** ........ 18
**Figure S16** ........ 19
**Figure S17** ........ 20
**Figure S18** ........ 21
**Figure S19** ........ 22
**Figure S20** ........ 23
**Figure S21** ........ 24
**Table S1** ........ 25
**Table S2** ........ 26
**Table S3** ........ 27
**Table S4** ........ 28

## Supplementary results

### *Optimization of the GSP-LFIA and AuNP-LFIA for HCG detection*

Several key factors, including the pH value (**Figures S4**), EDC concentration for covalent conjugation of antibody (**Figure S5A to S13A**), and saturated labeling amount of anti-HCG-β mAbs (**Figure S5B to S13B**), that affect the conjugation efficiency of antibody were systematically studied and optimized to obtain the best GSP or AuNP probes. Then, the concentration of anti-HCG-α mAbs sprayed on the T line (**Figure S15**), the GSP or AuNP probe amount used in each strip (**Figures S5C to S13C**), and the running strip time for signal readout (**Figures S5D to S13D**) were studied. The details for the optimal condition combinations that can enable the maximum $OD_T$ in the GSP-LFIA and AuNP-LFIA strips are summarized in **Table S2**.

### *Optimization of the GSP-LFIA and AuNP-LFIA for HBsAg detection*

To obtain the best detection sensitivity, various parameters that influence the sensitivity of $AuNP_{40}$-LFIA and $GSP_{270}$-LFIA strip, including saturated labeling amount of anti-HBsAg mAb (**Figures S19A** and **S20A**), concentration of anti-HBsAg pAb sprayed on the T line (**Figures S18B** and **S19B**), $GSP_{270}$ or $AuNP_{40}$ probe amount used in each strip (**Figures S19C** and **S20C**), and running strip time for signal readout (**Figures S18D** and **S20D**), were systematically investigated. The results show that the optimal combinations are as follows: saturated labeling amount of anti-HBsAg mAb of 0.38 mg/mL and 10.96 mg/mL, anti-HBsAg pAb of 3.0 mg/mL and 2.5 mg/mL, amount of $AuNP_{40}$ or $GSP_{270}$ probes in each strip of 5.325 fmol and 0.14 fmol for $AuNP_{40}$-LFIA and $GSP_{270}$-LFIA, respectively.

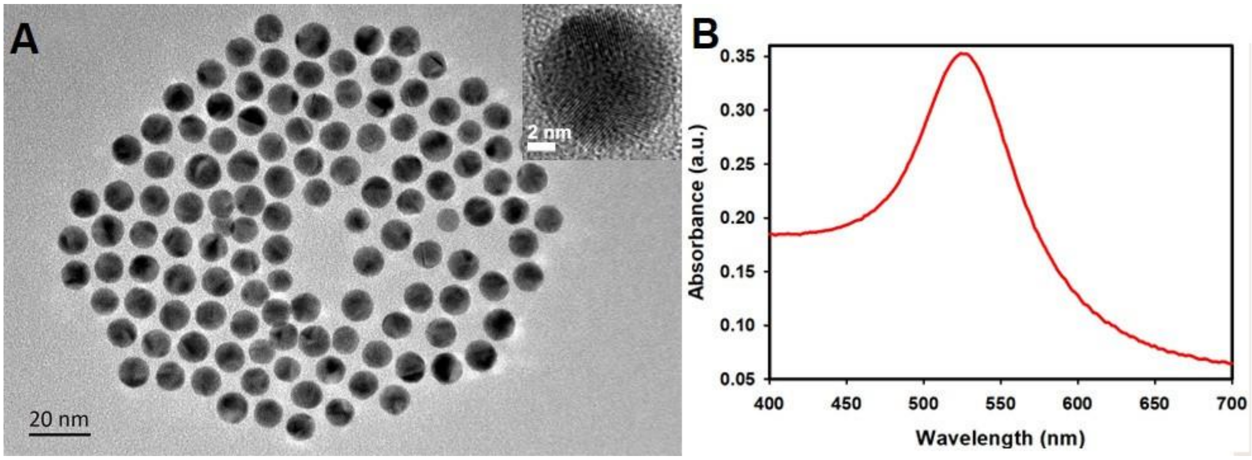


**Figure S1.** Characterization of oleylamine-coated AuNPs. (A) TEM image. (B) UV–vis absorption spectra. The maximum absorption peak of the hydrophobic AuNPs was located at 524 nm.

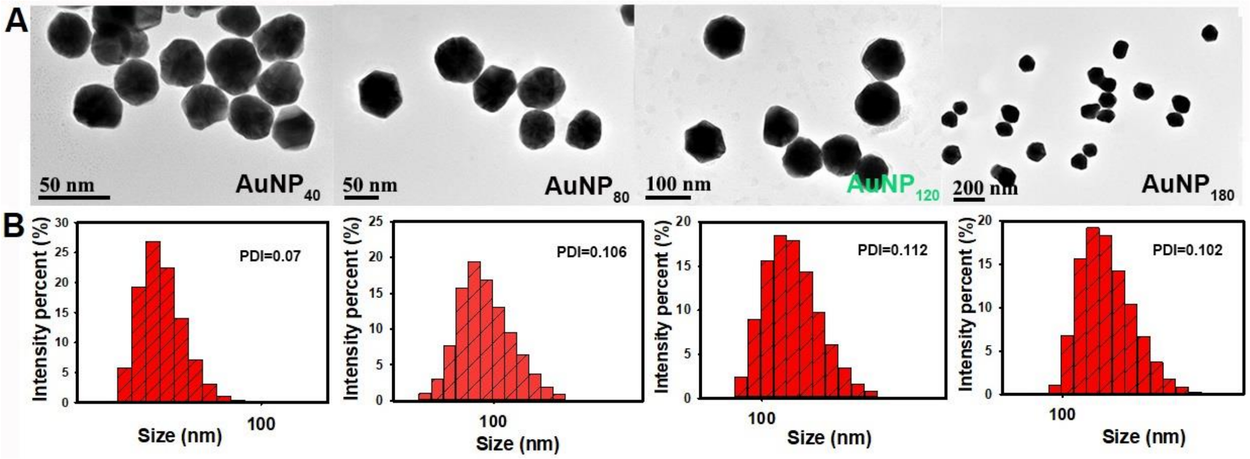


**Figure S2.** Characterization of different sized-AuNPs. (A) TEM images. (B) DLS analysis. For Figure S2A and S2B, from left to right: $AuNP_{40}$, $AuNP_{80}$, $AuNP_{120}$, and $AuNP_{180,}$ respectively.

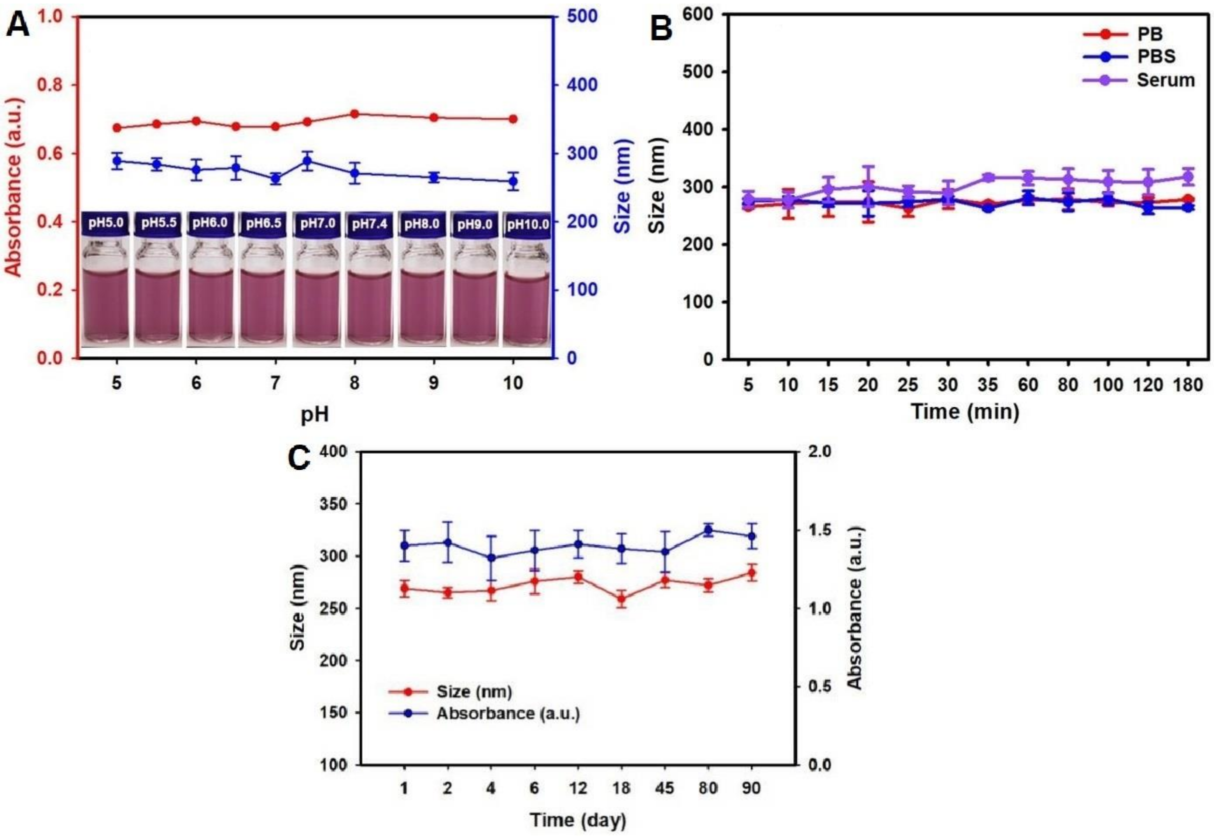


**Figure S3.** (A) Absorbance and hydrodynamic diameter of $GSP_{270}$ solutions under diverse pH values. The inset reveals the photograph of $GSP_{270}$ solutions under different pH values. (B) Hydrodynamic diameter variations of $GSP_{270}$ dispersed in PB, PBS, and serum against incubation time. (C) Evaluation of the long-term storage stability of GSPs by recording the changes in hydrodynamic diameter and absorbance of GSPs against 90-day storage.

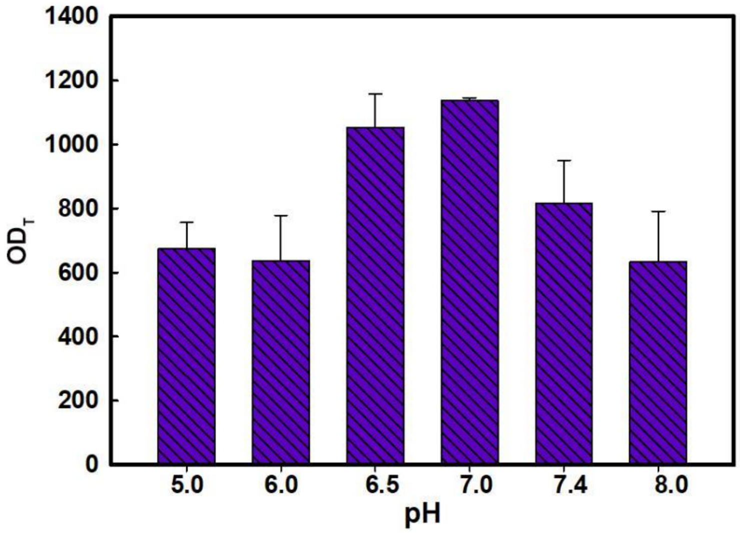


**Figure S4.** Effect of pH for the conjugation of anti-HCG-β mAb to GSPs or AuNPs.

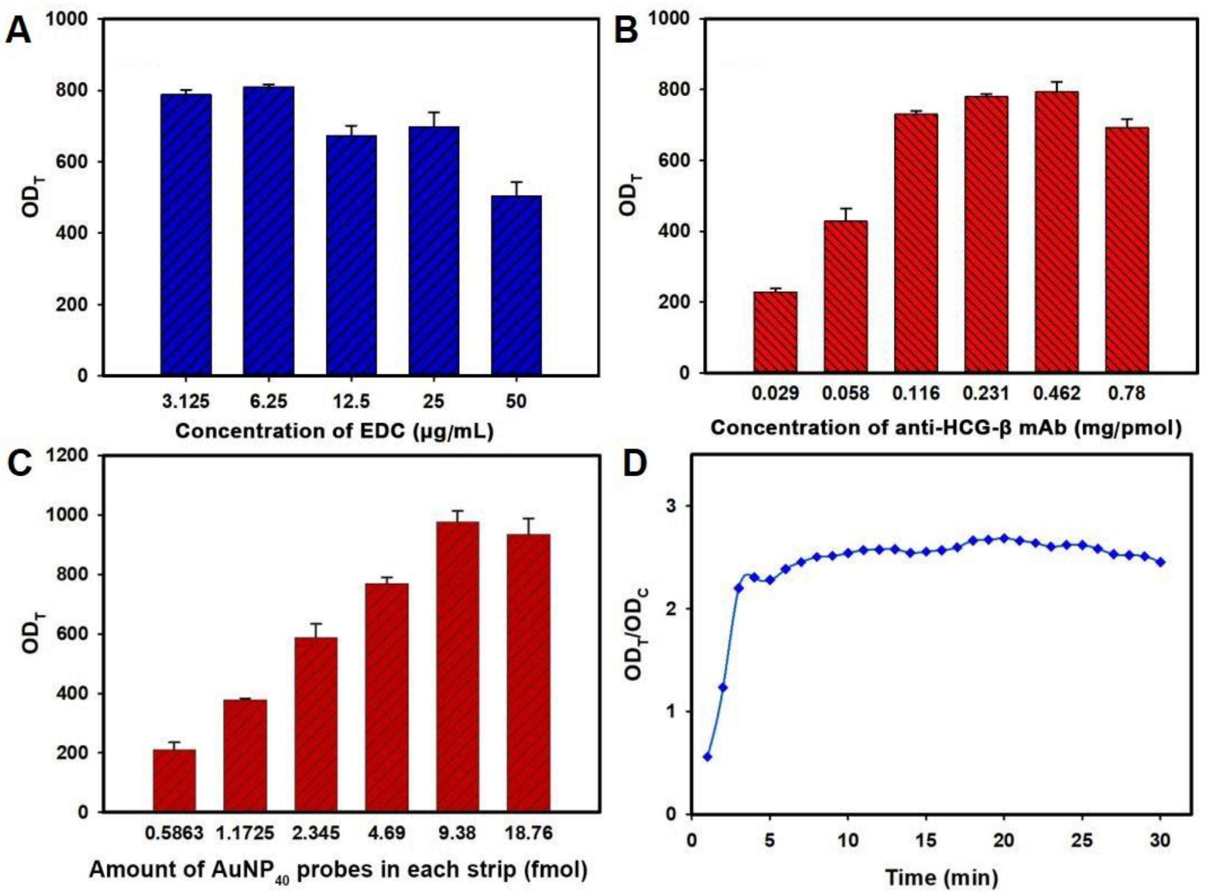


**Figure S5.** Parameter optimization of $AuNP_{40}$-LFIA for HCG detection. (A) The EDC concentration for the anti-HCG-β mAb conjugation. (B) The saturated labeling amount of anti-HCG-β mAb on the $AuNP_{40}$ surface. (C) The used amount of $AuNP_{40}$ probe in each strip. (D) The optimal readout time after running the strip with the sample solution.

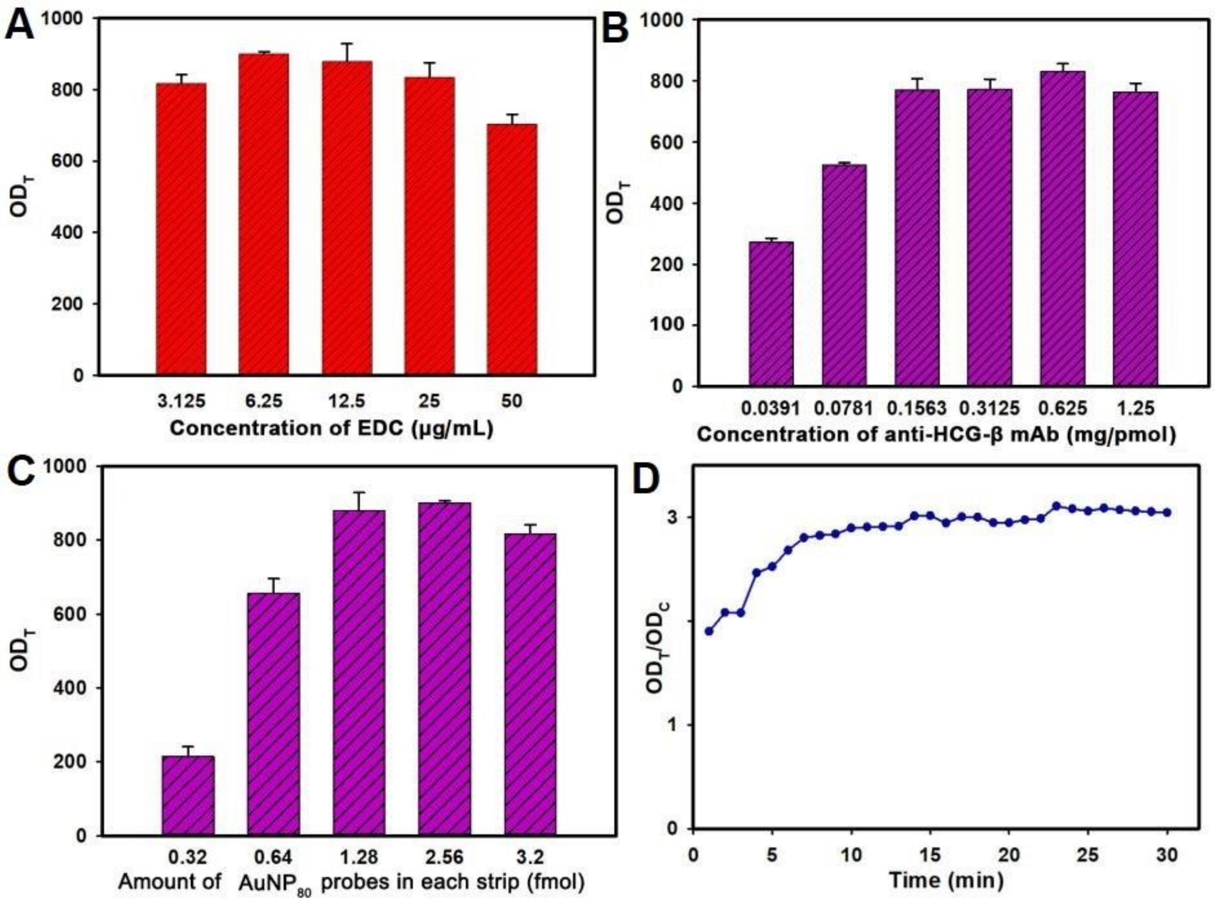


**Figure S6.** Parameter optimization of $AuNP_{80}$-LFIA for HCG detection. (A) The EDC concentration for the anti-HCG-β mAb conjugation. (B) The saturated labeling amount of anti-HCG-β mAb on the $AuNP_{80}$ surface. (C) The used amount of $AuNP_{80}$ probe in each strip. (D) The optimal readout time after running the strip with the sample solution.

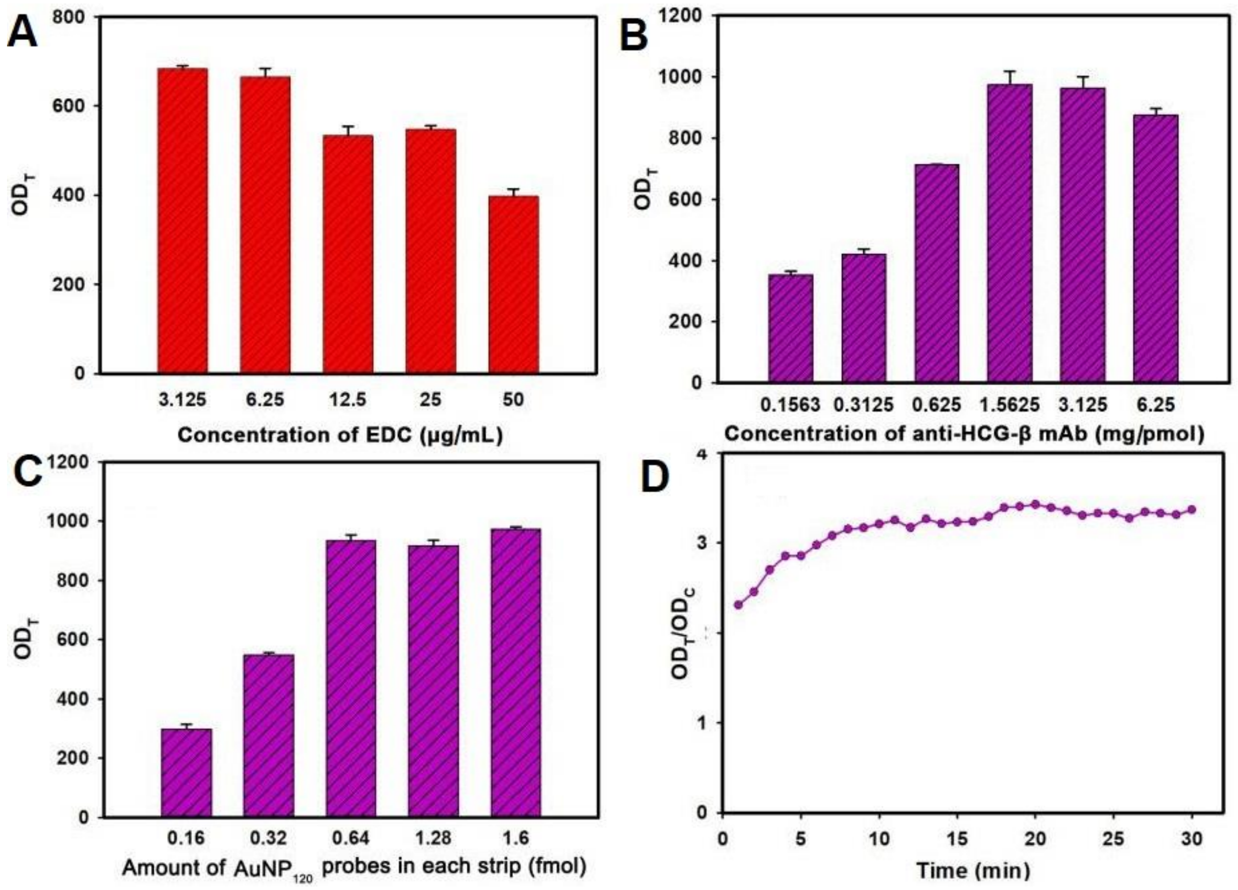


**Figure S7.** Parameter optimization of $AuNP_{120}$-LFIA for HCG detection. (A) The EDC concentration for the anti-HCG-β mAb conjugation. (B) The saturated labeling amount of anti-HCG-β mAb on the $AuNP_{120}$ surface. (C) The used amount of $AuNP_{120}$ probe in each strip. (D) The optimal readout time after running the strip with the sample solution.

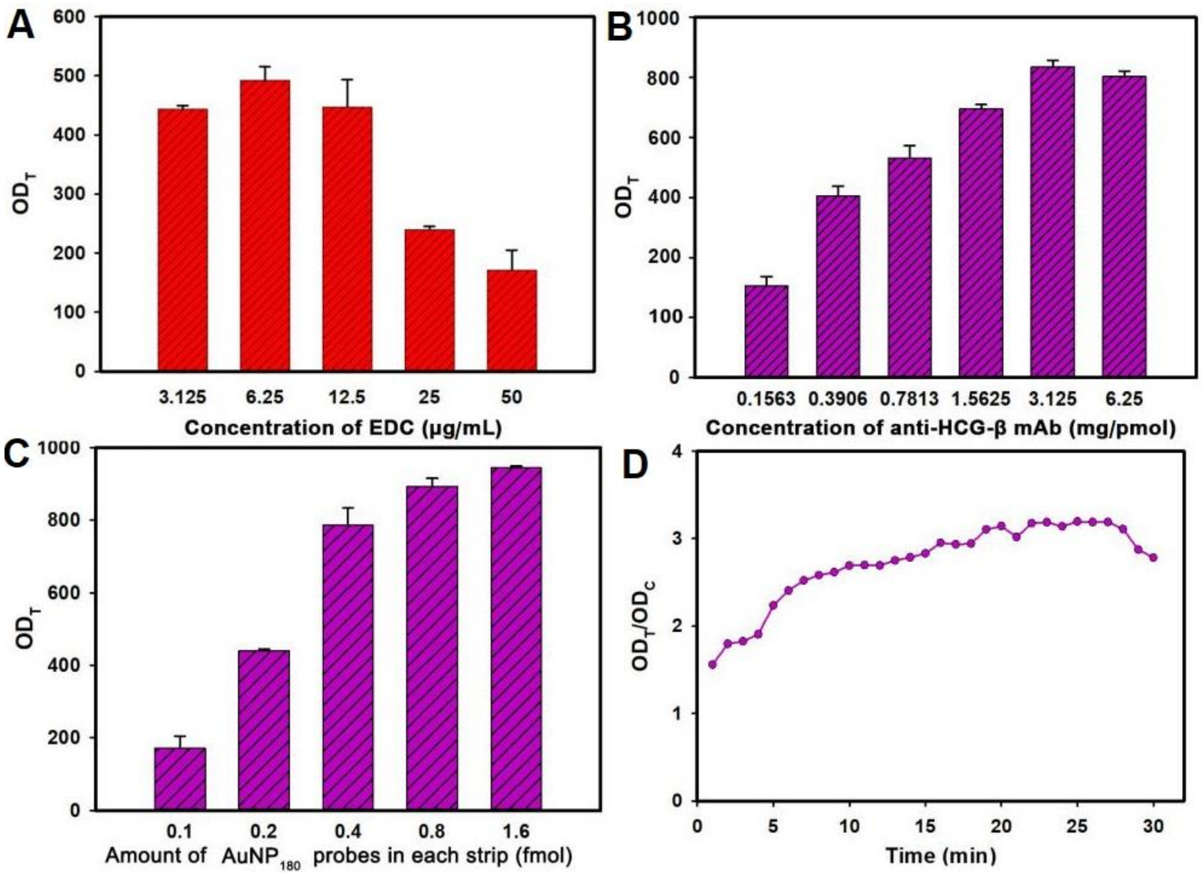


**Figure S8.** Parameter optimization of $AuNP_{180}$-LFIA for HCG detection. (A) The EDC concentration for the anti-HCG-β mAb conjugation. (B) The saturated labeling amount of anti-HCG-β mAb on the $AuNP_{180}$ surface. (C) The used amount of AuNP180 probe in each strip. (D) The optimal readout time after running the strip with the sample solution.

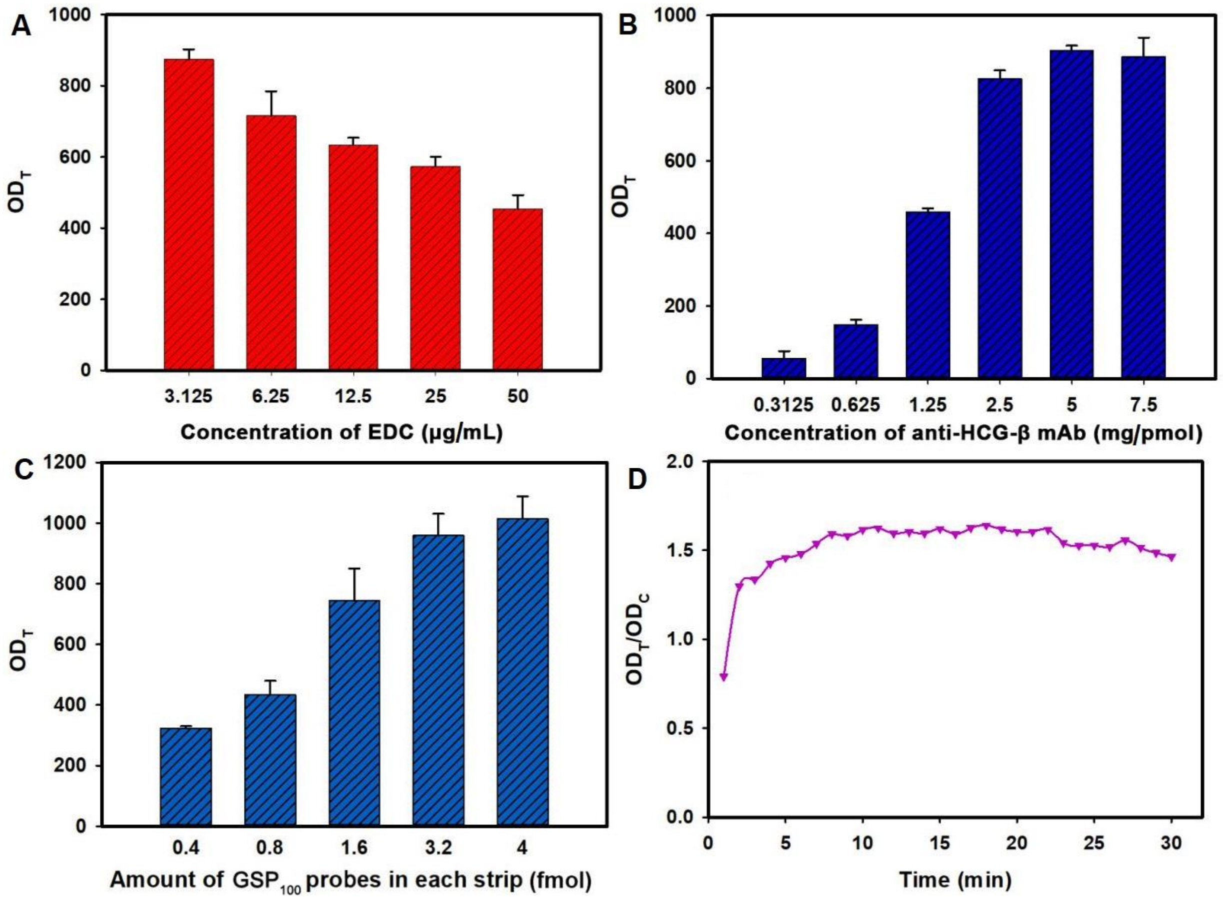


**Figure S9.** Parameter optimization of $GSP_{100}$-LFIA for HCG detection. (A) The EDC concentration for the anti-HCG-β mAb conjugation. (B) The saturated labeling amount of anti-HCG-β mAb on the $GSP_{100}$ surface. (C) The used amount of $GSP_{100}$ probe in each strip. (D) The optimal readout time after running the strip with the sample solution.

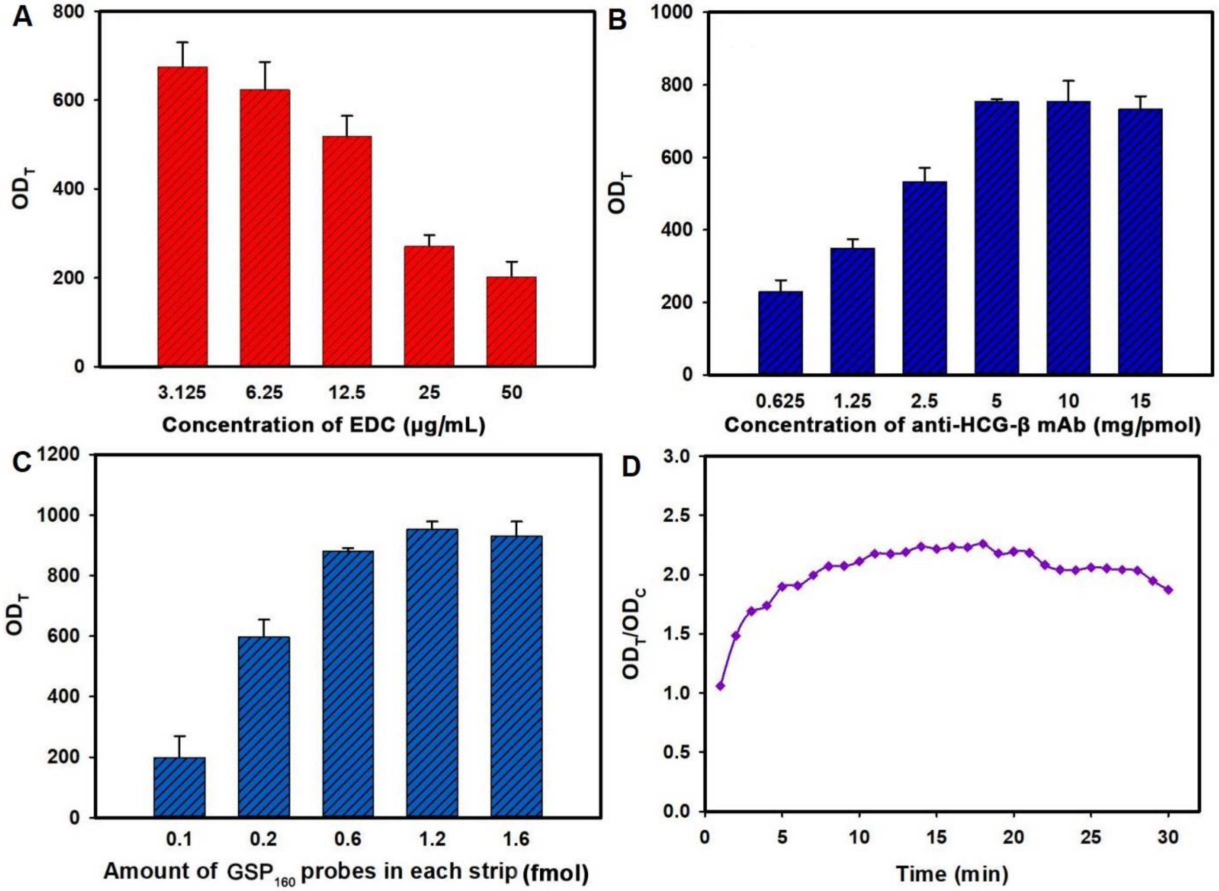


**Figure S10.** Parameter optimization of $GSP_{160}$-LFIA for HCG detection. (A) The EDC concentration for the anti-HCG-β mAb conjugation. (B) The saturated labeling amount of anti-HCG-β mAb on the $GSP_{160}$ surface. (C) The used amount of $GSP_{160}$ probe in each strip. (D) The optimal readout time after running the strip with the sample solution.

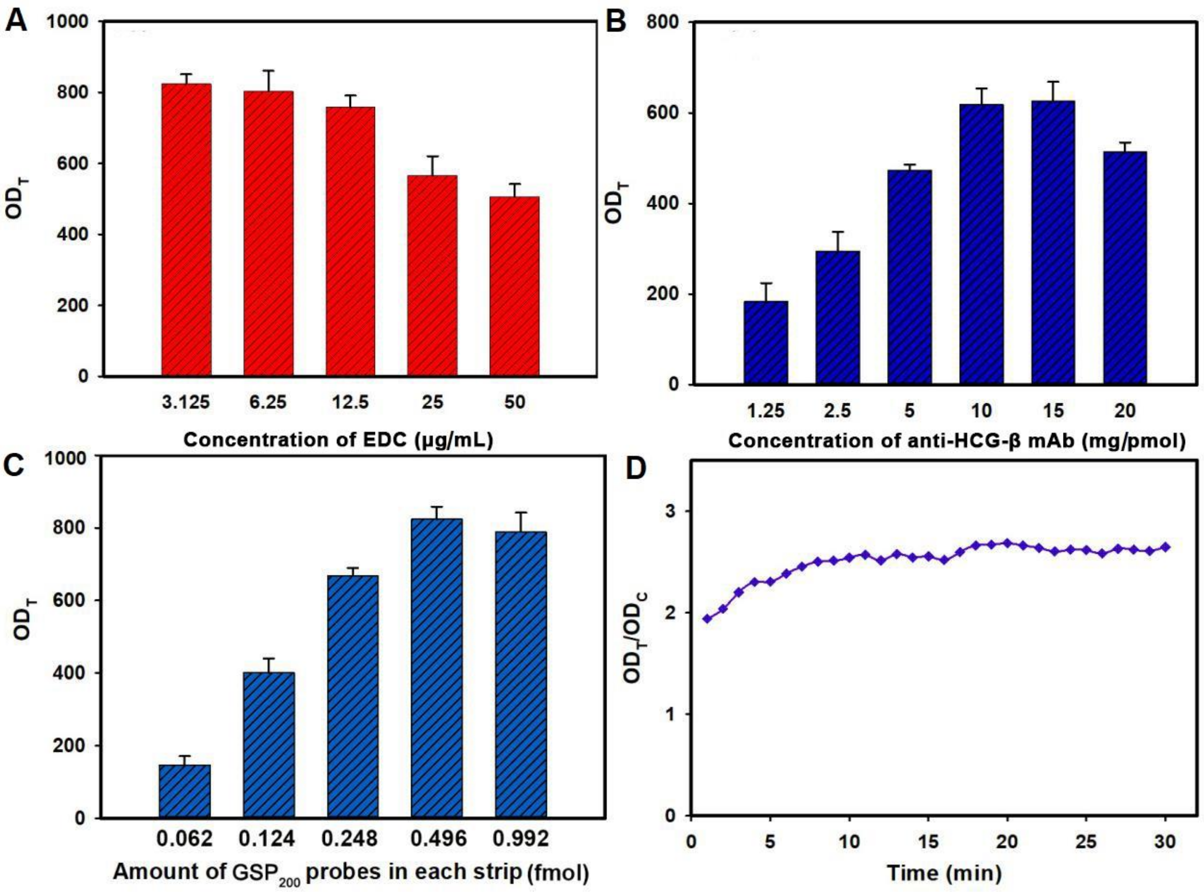


**Figure S11.** Parameter optimization of GSP$_{200}$-LFIA for HCG detection. (A) The EDC concentration for the anti-HCG-β mAb conjugation. (B) The saturated labeling amount of anti-HCG-β mAb on the GSP$_{200}$ surface. (C) The used amount of GSP$_{200}$ probe in each strip. (D) The optimal readout time after running the strip with the sample solution.

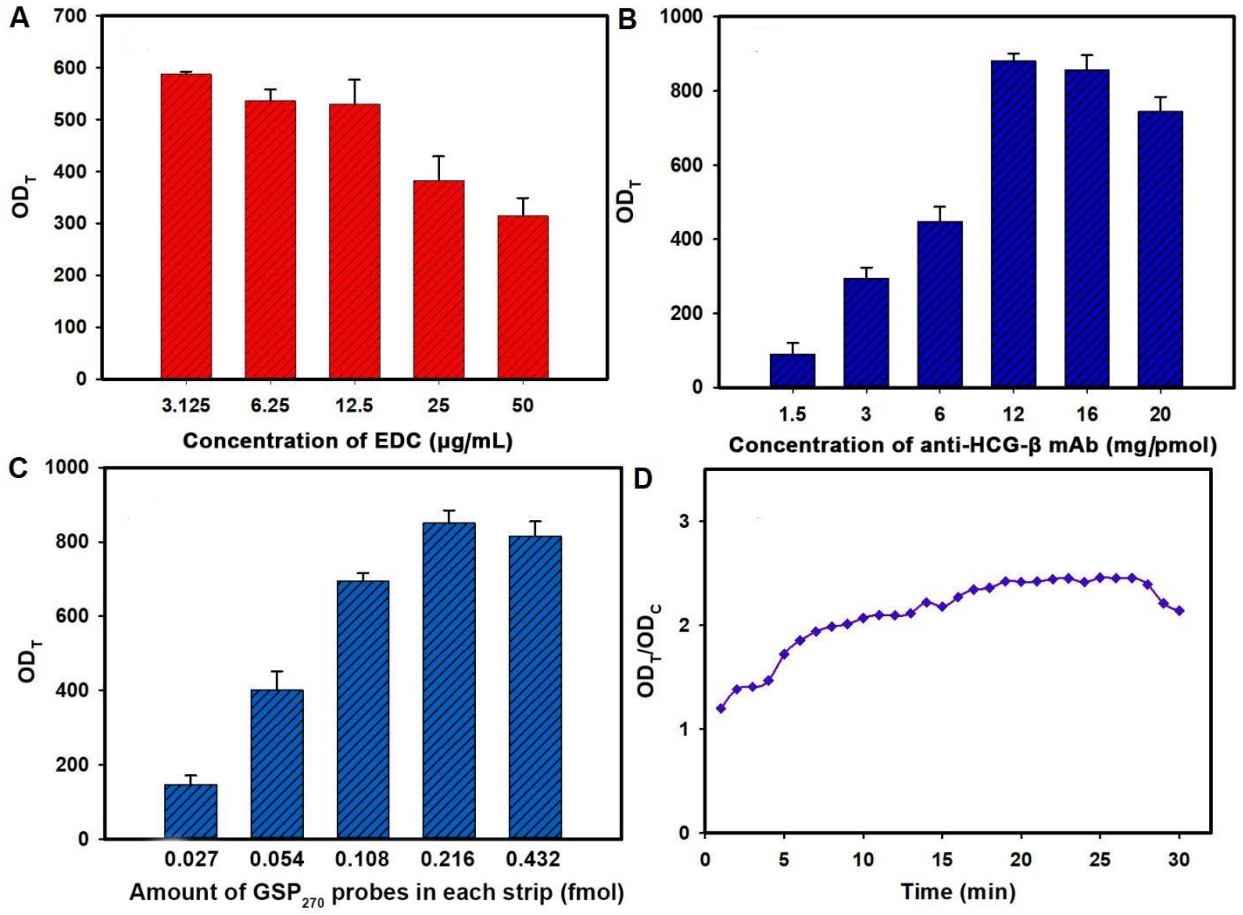


**Figure S12.** Parameter optimization of $GSP_{270}$-LFIA for HCG detection. (A) The EDC concentration for the anti-HCG-β mAb conjugation. (B) The saturated labeling amount of anti-HCG-β mAb on the $GSP_{270}$ surface. (C) The used amount of $GSP_{270}$ probe in each strip. (D) The optimal readout time after running the strip with the sample solution.

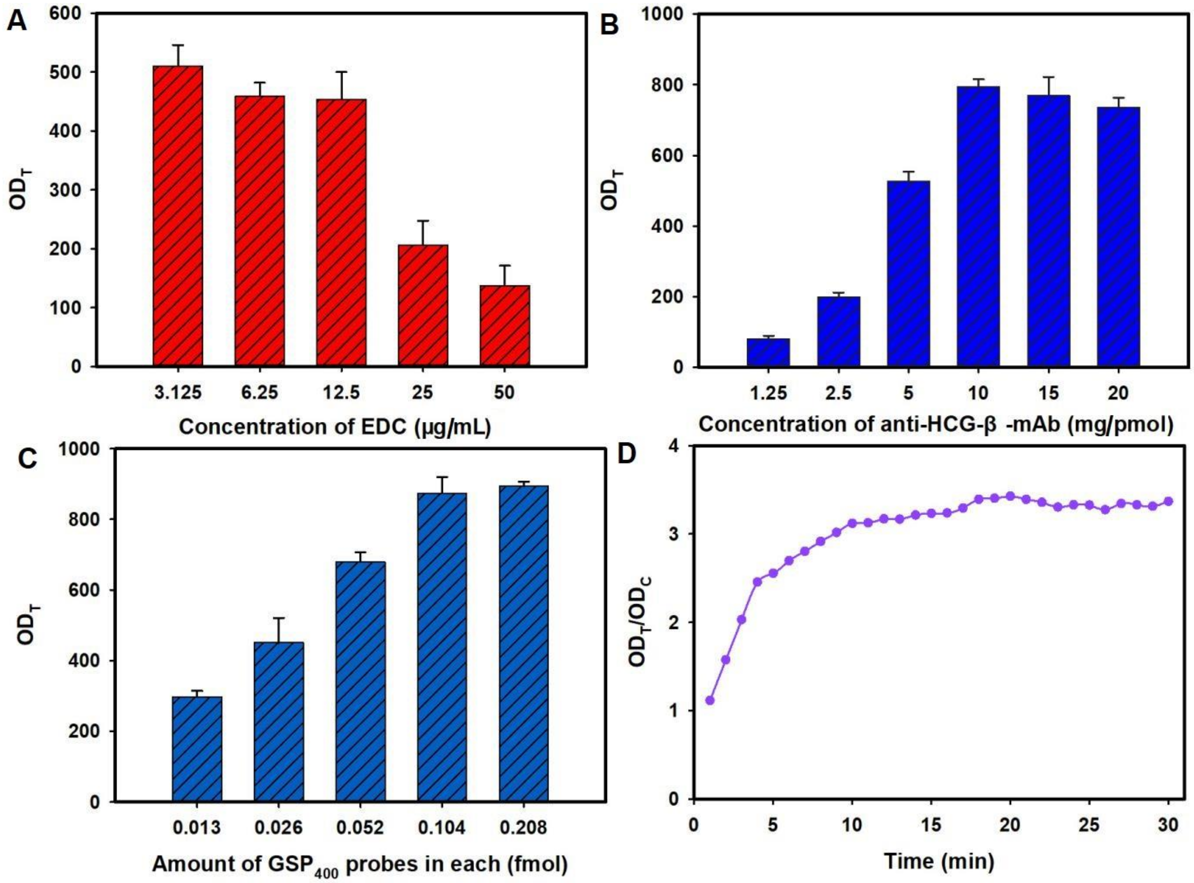


**Figure S13.** Parameter optimization of $GSP_{400}$-LFIA for HCG detection. (A) The EDC concentration for the anti-HCG-β mAb conjugation. (B) The saturated labeling amount of anti-HCG-β mAb on the $GSP_{400}$ surface. (C) The used amount of $GSP_{400}$ probe in each strip. (D) The optimal readout time after running the strip with the sample solution.

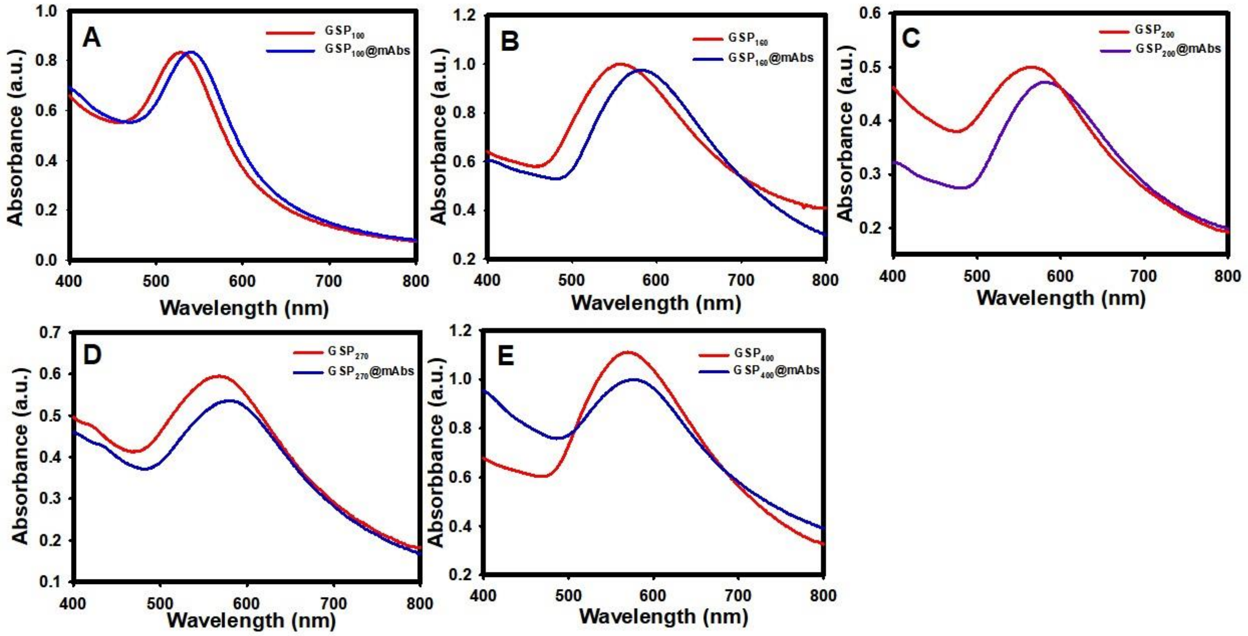


**Figure S14.** UV-vis absorption spectra for confirming the successful conjugation of GSPs with anti-HCG-α mAb. (A) $GSP_{100}$, (B) $GSP_{160}$, (C) $GSP_{200}$, (D) $GSP_{270}$, and (E) $GSP_{400}$.

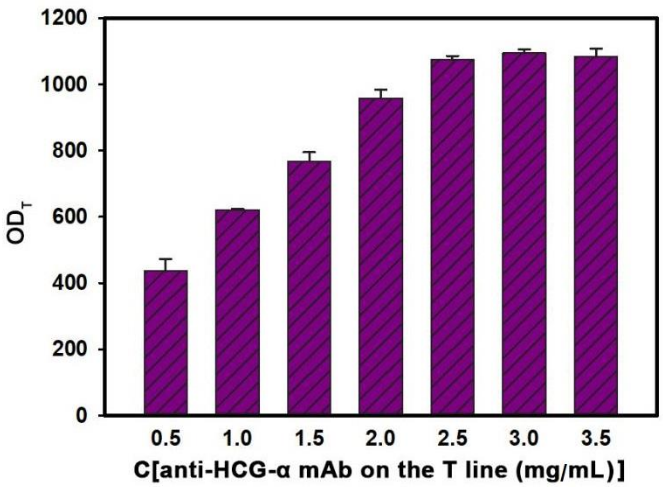


**Figure S15.** The concentration optimization of anti-HCG-α mAb sprayed on the T line of strip.

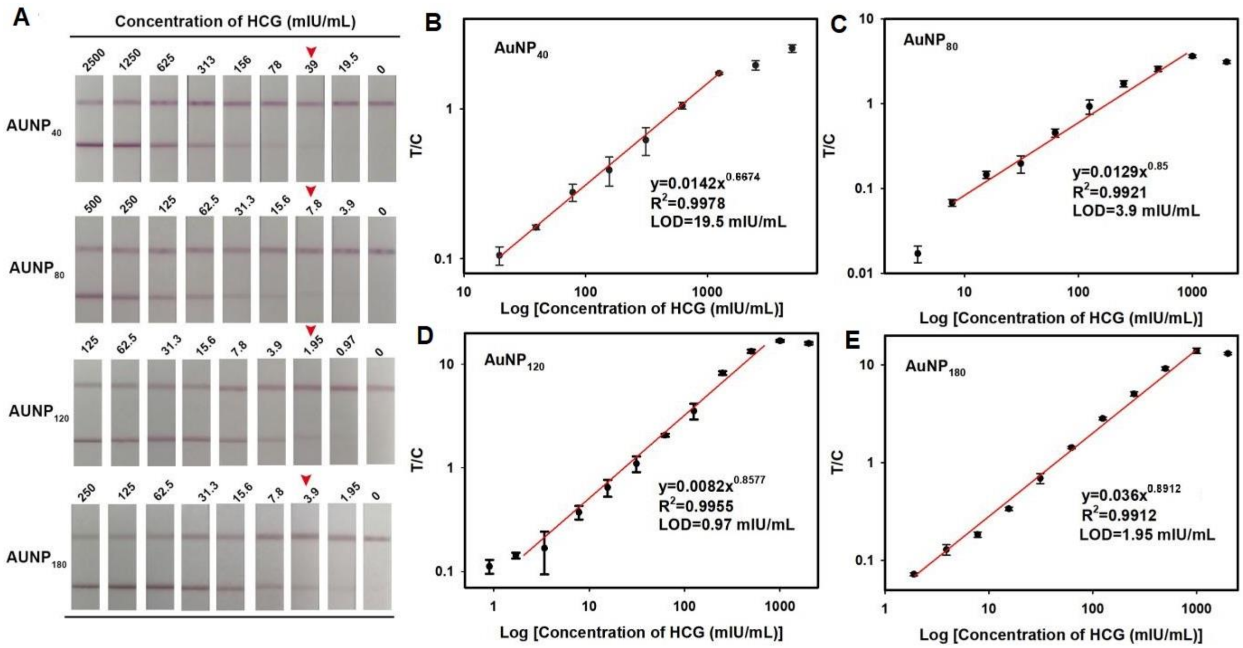


**Figure S16.** Qualitative and quantitative assay for HCG in serum using AuNP-LFIA. (A) The prototypes of four AuNP-LFIA strips responding to varying HCG concentrations. (B-E) Linear dependences against HCG concentrations of four AuNP-LFIA strips.

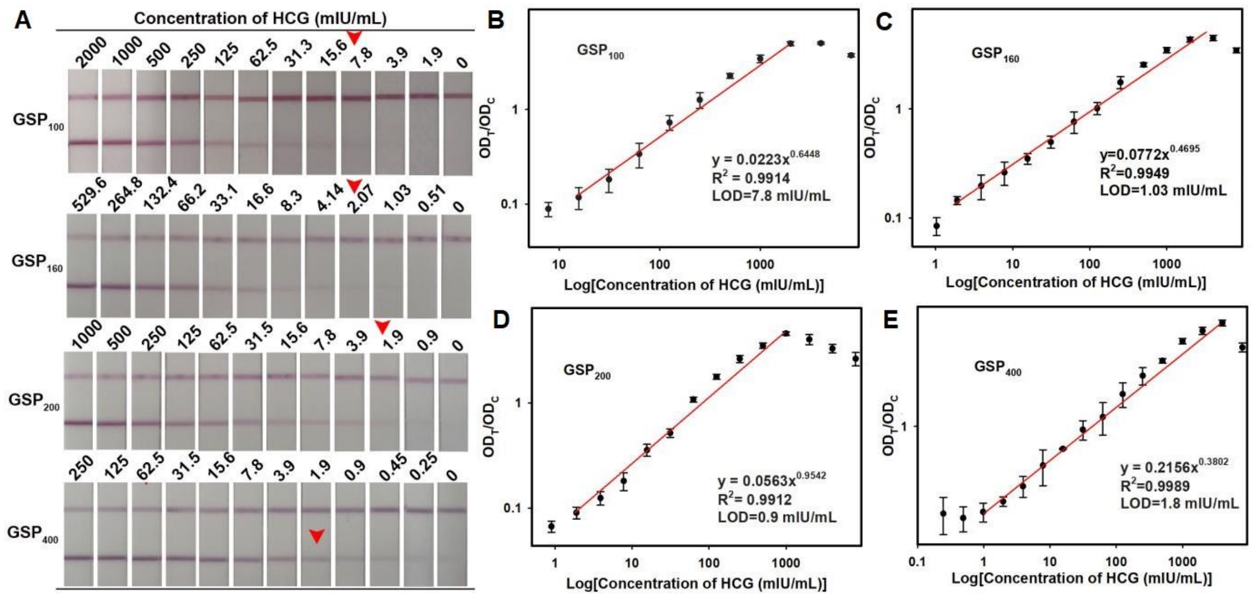


**Figure S17.** Qualitative and quantitative assay for HCG in serum using GSP-LFIA. (A) The prototypes of four GSP-LFIA strips responding to varying HCG concentrations. (B-E) Linear dependences against HCG concentrations of four GSP-LFIA strips.

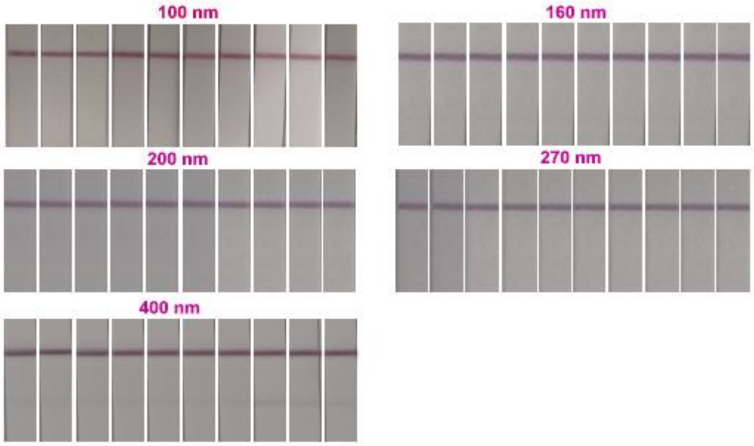


**Figure S18.** The strip prototypes from the detection of ten HCG-negative serum samples using five different GSP-LFIA strips, respectively. From these pictures, we can see that obvious background bands at the T zones when we used the $GSP_{400}$-LFIA strip to detect ten blank samples, whereas no background signal was seen with other four GSP-LFIA strips, confirming the fact that 400 nm partly settled in the test area of NC membrane to form background value even in the absence of targets.

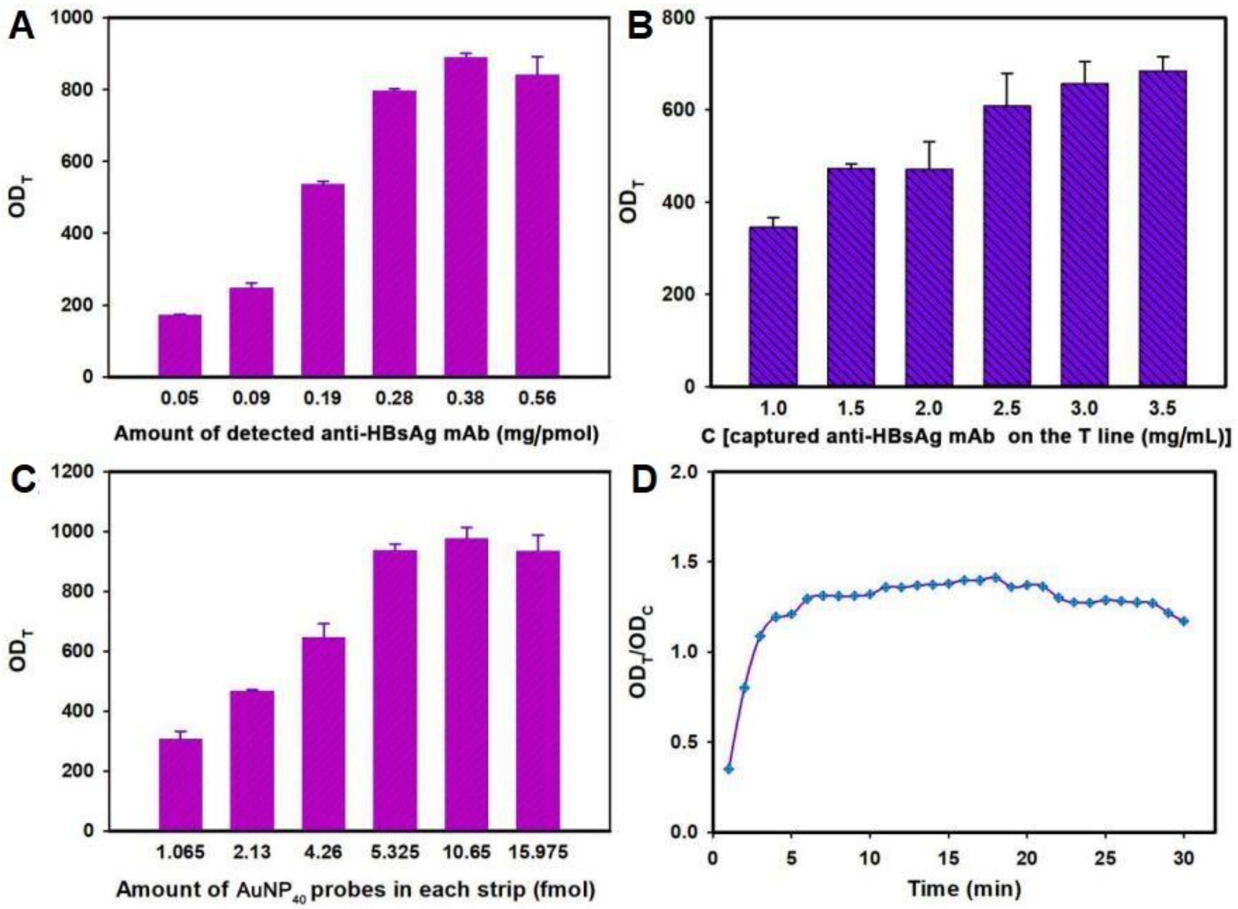


**Figure S19.** Parameter optimization of $AuNP_{40}$-LFIA for HBsAg detection. (A) The saturated labeling amount of detected anti-HBsAg mAb on the $AuNP_{40}$ surface. (B) The concentration of captured anti-HBsAg mAb sprayed on the T line. (C) The used amount of $AuNP_{40}$ probe in each strip. (D) The optimal readout time after running the strip with the sample solution.

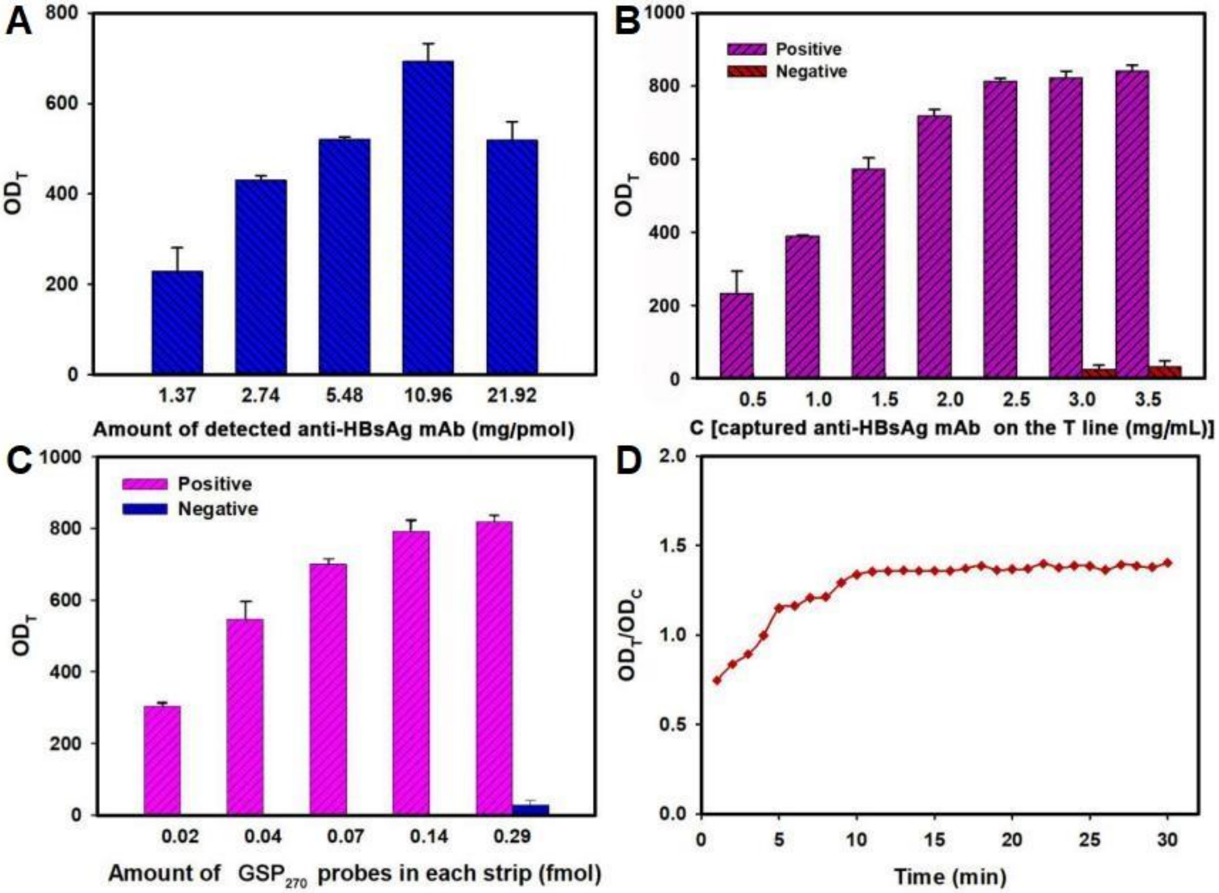


**Figure S20.** Parameter optimization of $GSP_{270}$-LFIA for HBsAg detection. (A) The saturated labeling amount of detected anti-HBsAg mAb on the $GSP_{270}$ surface. (B) The concentration of captured anti-HBsAg mAb sprayed on the T line. (C) The used amount of $GSP_{270}$ probe in each strip. (D) The optimal readout time after running the strip with the sample solution.

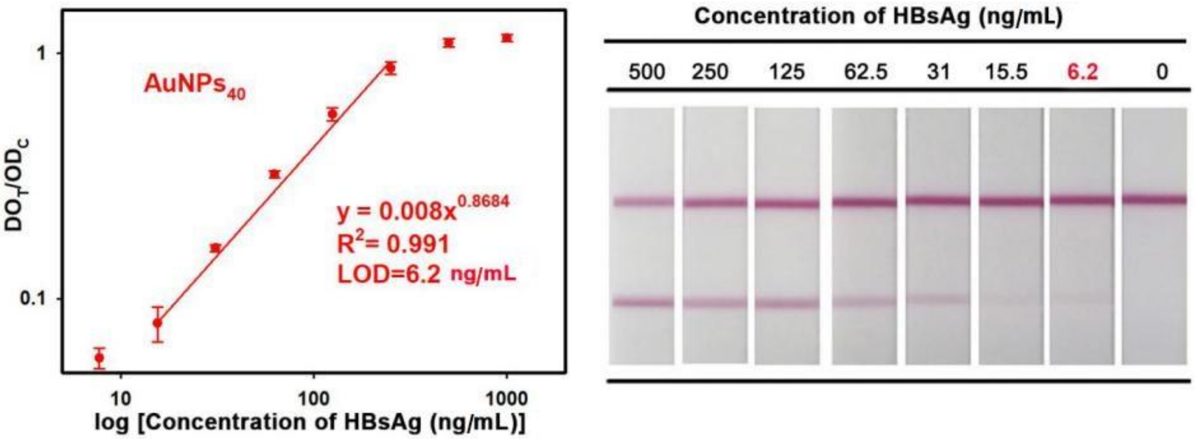


**Figure S21.** $AuNP_{40}$-LFIA strips for HBsAg detection in serum.

**Table S1.** Synthesis conditions of different GSPs.

| Size (nm) | Oleylamine-coated AuNPs (mg) | PMAO (mg) | SDS (mg) | Oil/water (μL/μL) | Ultrasonic power (960 W) |
|---|---|---|---|---|---|
| 100 nm | 10 | 0.5 | 6 | 50/500 | 8% |
| 160 nm | 10 | 0.5 | 4 | 50/500 | 8% |
| 200 nm | 10 | 0.5 | 2.5 | 20/500 | 10% |
| 270 nm | 10 | 0.5 | 2.5 | 20/500 | 16% |
| 400 nm | 10 | 0.5 | 2.5 | 20/500 | 20% |

**Table S2.** The optimal experimental condition combinations used for HCG detection with GSP-LFIA or AuNP-LFIA.

| Labels | pH | The EDC concentration (μg/mL) | The saturated labeling amount of anti-HCG-β mAbs (mg/pmol) | Tapture mAbs sprayed on the T line (mg/mL) | The GSP or AuNP probe amount used in each strip (fmol) | The running time for signal readout (min) |
|---|---|---|---|---|---|---|
| $AuNP_{40}$ | 7 | 3.125 | 0.321 | 2.5 | 6.23 | 15 |
| $AuNP_{80}$ | 7 | 3.125 | 0.625 | 2.5 | 1.28 | 15 |
| $AuNP_{120}$ | 7 | 3.125 | 1.5625 | 2.5 | 0.64 | 15 |
| $AuNP_{180}$ | 7 | 6.25 | 3.125 | 2.5 | 0.8 | 20 |
| $GSP_{100}$ | 7 | 3.125 | 5 | 2.5 | 3.2 | 15 |
| $GSP_{160}$ | 7 | 3.125 | 5 | 2.5 | 1.2 | 15 |
| $GSP_{200}$ | 7 | 3.125 | 10 | 2.5 | 0.496 | 15 |
| $GSP_{270}$ | 7 | 3.125 | 12 | 2.5 | 0.216 | 15 |
| $GSP_{400}$ | 7 | 3.125 | 10 | 2.5 | 0.104 | 15 |

**Table S3.** Correlation analysis for HBsAg detection in serum among three methods, including $GSP_{270}$-LFIA, $AuNP_{40}$-LFIA, and the clinically well-accepted CLIA kits.

| Sample | $GSP_{270}$-LFIA | | $AuNP_{40}$-LFIA | | CLIA |
|---|---|---|---|---|---|
| | Detected concentration (ng/mL) | CV (%) | Detected concentration (ng/mL) | CV (%) | Detected concentration (ng/mL) |
| 1 | 133.82 ±7.35 | 5.49 | 111±22.8 | 20.54 | 156.6 |
| 2 | 120.45 ±4.17 | 3.46 | 141±15.66 | 11.10 | 147.3 |
| 3 | 88.87±1.90 | 2.13 | 80±14.24 | 17.8 | 99.2 |
| 4 | 83.09 ±5.64 | 6.79 | 90.6±6.51 | 7.18 | 102.3 |
| 5 | 62.34 ±2.47 | 3.96 | 42.5±13.44 | 31.62 | 78.4 |
| 6 | 57.90 ±6.59 | 11.38 | 75±24.24 | 32.32 | 60.5 |
| 7 | 114.28 ±1.92 | 3.35 | 106.05±14.21 | 13.39 | 121.08 |
| 8 | 48.62 ±1.89 | 3.89 | 33.23±9.53 | 28.67 | 58.3 |
| 9 | 38.43 ±3.17 | 8.26 | 36.5±4.95 | 13.56 | 51.2 |
| 10 | 25.51 ±1.21 | 4.75 | 23.15±20.11 | 86.87 | 30.25 |
| 11 | 208.4 ±19.7 | 9.45 | 197.34±6.25 | 3.17 | 219 |
| 12 | 200.2 ±13.7 | 6.83 | 254.35±23.64 | 9.297 | 224 |
| 13 | 18.37 ±1.75 | 9.53 | 19.45±14.61 | 75.11 | 23.4 |
| 14 | 18.09 ±1.40 | 7.75 | 12.35±4.74 | 38.38 | 25.0 |
| 15 | 17.53 ±0.79 | 4.53 | 16.5±1.78 | 10.78 | 20.0 |
| 16 | 15.29 ±0.87 | 5.71 | 23.75±1.20 | 5.05 | 16.3 |
| 17 | 153±0.61 | 4.02 | 149.42±3.65 | 2.44 | 163.3 |
| 18 | 12.55 ±0.98 | 7.80 | 9.7±0.67 | 6.91 | 18.32 |
| 19 | 7.54 ±1.01 | 13.41 | 13.2±1.18 | 8.94 | 10.0 |
| 20 | 6.32 ±0.82 | 13.06 | 9.55±0.16 | 1.68 | 8.0 |
| 21 | 66.25 ±8.77 | 13.23 | 64.34±8.24 | 12.81 | 75 |
| 22 | 86.02 ±7.01 | 8.15 | 67.35±5.34 | 7.93 | 100 |
| 23 | 4.90±0.22 | 4.49 | ND | | 5.0 |
| 24 | 78.61±0.67 | 14.44 | 68.33±9.43 | 13.81 | 75.3 |
| 25 | 3.45 ±1.08 | 31.37 | 6.53±1.23 | 18.86 | 6.0 |
| 26 | 3.13 ±0.19 | 6.07 | ND | | 4.5 |
| 27 | 2.74 ±0.20 | 7.44 | ND | | 4.0 |
| 28 | 30.02±0.42 | 20.57 | 39.63±5.77 | 14.56 | 45.5 |
| 29 | 2.02 ±0.08 | 3.88 | ND | | 3.0 |
| 30 | 1.76 ±0.14 | 7.95 | ND | | 2.6 |
| 31 | 1.49 ±0.21 | 14.06 | ND | | 2.0 |
| 32 | 1.25 ±0.33 | 26.73 | ND | | 1.5 |
| 33 | 0.88 ±0.09 | 10.22 | ND | | 1.0 |
| 34 | 0.44 ±0.14 | 30.96 | ND | | 0.8 |
| 35 | 0.41 ±0.09 | 22.68 | ND | | 0.5 |
| 36 | ND | | ND | | ND |
| 37 | ND | | ND | | ND |
| 38 | ND | | ND | | ND |
| 39 | ND | | ND | | ND |
| 40 | ND | | ND | | ND |
| 41 | ND | | ND | | ND |
| 42 | ND | | ND | | ND |
| 43 | ND | | ND | | ND |
| 44 | ND | | ND | | ND |
| 45 | ND | | ND | | ND |

**Table S4** A comparison of the detection performance of our GSPs based LFIA and other reported gold-based immunoassay methods.

| Method | Signal output mechanism | Linear range | Limit of detection | Reference |
|---|---|---|---|---|
| GSPs based LFIA | GSPs | 0.46 ~1000 ng/mL | 0.46 ng/mL | This work |
| Capacitive immunosensor[1] | Planar gold nanoparticles | 10 ~ 60 ng/mL | 10 ng/mL | Anal. Methods 2013, 5, 4448 |
| Homogeneous fluorescence assay[2] | Europium-chelate-adsorbed silica nanoparticles | 10 ~ 200 ng/mL | 10 ng/mL | Anal. Methods 2012,4, 3810–3815. |
| Enhanced LFIA[3] | Dual gold nanoparticle conjugates | 0.1 ~ 30 ng/mL | 0.06 ng/mL | ACS Omega 2019, 4, 5083-5087 |
| Electrochemical immunoassay[4] | Antigen-antibody reaction combined with nanogold | 0.5 ~ 650 ng/mL | 0.1 ng/mL | Microchim. Acta. 2009, 166, 269–275. |
| Conductometric immunoassay[5] | Double-codified nanogold particles | 0.1 ~ 600 ng/mL | 0.01 ng/mL | Biochem. Eng. J. 2009, 45, 107–112. |
| Dynamic light scattering[6] | Target-induced aggregation of gold nanoparticles | 0.0051 IU/mL | 0.005 IU/mL | Anal. Biochem 2012, 428, 119–125 |
| Surface-enhanced Raman scattering (SERS)[7] | Gold nanoflower based SERS | 0.03 ~ 0.62 IU/mL | 0.01 IU/mL | Biosens. Bioelectron. 2015,66, 461–467 |
| Localized surface plasmon resonance (LSPR)[8] | Gold nanorod based LSPR | 0.01 ~ 1 IU/mL | 0.01 IU/mL | Biosens. Bioelectron. 2010, 26, 404–410 |

**Calculation of extinction Molar decadic extinction coefficient ε**

ε of AuNPs and GSPs were calculated according to the Lambert-Beer[9]:

$$\varepsilon = \frac{A}{CL} \quad (1)$$

where A is the UV–vis absorbance of the nanoparticle solution, and L is the path length of the measuring beam in the sample. C is the concentration of the nanoparticles. AuNPs with a size of 80nm, 120nm and 180 were synthesized following a kinetically controlled seeded growth strategy. Hence, $C_{AuNPs}$ was obtained from the concentration of seed gold, which was calculated according to a previous research.[10]

Furthermore, the $C_{GSPs}$ were calculated form the following formula:

$$C = \frac{(m_1\rho_2 + m_2\rho_1)\ P}{\frac{4}{3}\prod R^3 \rho_1 \rho_2 NAXV} \quad (2)$$

Where $m_1$ and $m_2$ is the adding quality of the oleylamine-coated AuNPs and the PMAO when synthesizing different sized of GSPs, respectively. The $\rho_1$ and $\rho_2$ are the densities of the Au and the PMAO, respectively. The R refers to the radius of GSPs, and V is the volume of the sample solution, NA is the Avogadro's number. P is the productive rate of GSPs, and where x is the dilution ratio of the measuring sample.

**Calculation of the number of internal oleylamine-coated AuNPs in each different sized GSP**

The number (N) of internal oleylamine-coated AuNPs in each different sized GSPs were estimated according to the following formula:

$$N = \frac{\rho_2 m_1 R_x{}^3}{(m_1\rho_2 + m_2 p_1) P R_0{}^3} \quad (3)$$

Where Rx and $R_0$ is the radius of GSPs and the oleylamine-coated AuNPs, respectively.

## References


1. Alipour E, Ghourchian H, Boutorabi S M. Gold nanoparticle based capacitive immunosensor for detection of hepatitis B surface antigen. Anal Methods. 2013; 5 (17):4448-53.
2. Dou XR, Wu ZZ, Hu ZY, Zhu XT, Xu R, Xie L, et al. Preparation of immuno-probes based on europium-chelate-adsorbed silica nanoparticles and magnetic nanoparticles and their application in detection of hepatitis B surface antigen. Anal Methods. 2012; 4: 3810-5.
3. Shen Y, Shen G. Signal-enhanced lateral flow immunoassay with dual gold nanoparticle conjugates for the detection of hepatitis B surface antigen. ACS Omega. 2019; 4: 5083-7.
4. Wu S, Zhong Z, Wang D, Li M, Qing Y, Dai N, et al. Gold nanoparticle-labeled detection antibodies for use in an enhanced electrochemical immunoassay of hepatitis B surface antigen in human serum. Microchim Acta. 2009; 166: 269-75.
5. Liu H, Yang Y, Chen P, Zhong Z. Enhanced conductometric immunoassay for hepatitis B surface antigen using double-codified nanogold particles as labels. Biochem Eng J. 2009: 45: 107-112.
6. Wang X, Li Y, Quan D, Wang J, Zhang Y, Du J, Peng J, Fu Q, Zhou Y, Jia S. Detection of hepatitis B surface antigen by target-induced aggregation monitored by dynamic light scattering. Anal. Biochem. 2012; 428: 119-25.
7. Kamińska A, Witkowska E, Winkler K, Dzięcielewski I, Weyher J L, Waluk J. Detection of Hepatitis B virus antigen from human blood: SERS immunoassay in a microfluidic system. Biosens Bioelectron. 2015; 66: 461-7.
8. Wang X, Li Y, Wang H, Fu Q, Peng J, Wang Y, Du J, Zhou Y, Zhan L. Gold nanorod-based localized surface plasmon resonance biosensor for sensitive detection of hepatitis B virus in buffer, blood serum and plasma. Biosens Bioelectron. 2010; 26: 404-10.
9. Mäntele W, Deniz E, UV–vis absorption spectroscopy: lambert-beer reloaded. Spectrochim. Acta Part A. 2017;173: 965-8.
10. Haiss W,Thanh NT, Aveyard J, Fernig DG, Determination of size and concentration of gold nanoparticles from UV-vis spectra. Anal Chem. 2007; 79: 4215-21.
